# Revisiting the genus *Bolbosoma* Porta, 1908 (Acanthocephala: Polymorphidae): host specificity, phylogeny, and species synonymization

**DOI:** 10.1186/s13071-025-07015-3

**Published:** 2025-09-24

**Authors:** Alicia García-Gallego, Francisco J. Aznar, Jesús S. Hernández-Orts, Nicholas J. Davison, Andrew G. Briscoe, Rocío Loizaga, D. Timothy J. Littlewood, Natalia Fraija-Fernández

**Affiliations:** 1 Marine Zoology Unit, Cavanilles Institute of Biodiversity and Evolutionary Biology, University of Valencia, Valencia, Spain; 2 Natural History Museum, London, UK; 3 Institute of Parasitology, Biology Centre, Czech Academy of Sciences, České Budějovice, Czech Republic; 4 Scottish Marine Animal Stranding Scheme, School of Biodiversity, One Health and Veterinary Medicine College of Medical, Veterinary and Life Sciences University of Glasgow, Glasgow, G12 8QQ UK; 5 Centro Para el Estudio de Sistemas Marinos-Consejo Nacional de Investigaciones Científicas y Técnicas, Puerto Madryn, Argentina

**Keywords:** Acanthocephala, *Bolbosoma*, Cetaceans, Host-specificity, Systematics, Mitogenome, 18S rDNA, 28S rDNA

## Abstract

**Background:**

Acanthocephalans of the genus *Bolbosoma* Porta, 1908 are trophically transmitted parasites that infect marine mammals (mostly cetaceans and less frequently pinnipeds) worldwide. There are 12 species currently considered as valid; however, most records lack information on the maturity stage of the specimens. This, coupled with the scarce phylogenetic information available, hinders a correct understanding of their patterns of host specificity, evolutionary history, and taxonomy. A particularly intriguing case is that of *Bolbosoma vasculosum* (Rudolphi, 1819), which has been frequently reported in odontocetes but rarely as an adult, having been suggested to be synonymous with *Bolbosoma capitatum* (von Linstow, 1880).

**Methods:**

We used a comprehensive approach to investigate the concept of *Bolbosoma*. First, we conducted a bibliographic review of records of *Bolbosoma* spp. to clarify which are the final hosts for each species. We paid particular attention to *B. vasculosum*, using morphological and molecular analyses to compare it with *B. capitatum*. Second, we characterized the complete mitochondrial genome of *Bolbosoma balaenae* (Gmelin, 1790), *Bolbosoma turbinella* (Diesing, 1851), *B. capitatum*, and *B. vasculosum*. Then, we reconstructed the phylogenetic relationships of *Bolbosoma* spp. and related taxa using full mitochondrial genomes (or only *cox1* when full mitogenomes were unavailable) and nuclear ribosomal genes (18S and 28S).

**Results:**

*Bolbosoma* spp. exhibit high specificity for cetaceans, with no confirmed records of adult specimens in other host groups. Within this genus, *B. vasculosum* appears to be conspecific with *B. capitatum* based on both morphological and molecular evidence. This species shows high affinity to odontocetes, while the remaining species are specific to mysticetes. Phylogenetic analyses showed strong support for the monophyly of *Bolbosoma* spp., which appeared as sister taxa to *Corynosoma* spp. and *Andracantha* spp.. The resulting topology aligns with the patterns of specificity indicated by host records, revealing two distinct clades for species specific to odontocetes and mysticetes, respectively.

**Conclusions:**

The phylogenetic relationships obtained support the hypothesis that the association of *Bolbosoma* spp. with cetaceans originated through a host-switching event from aquatic birds.

**Figure:**
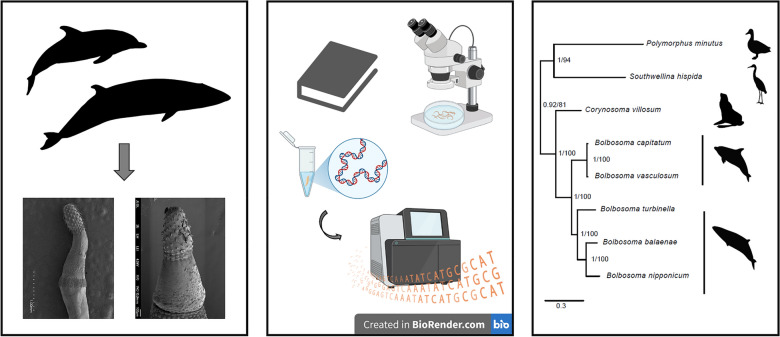

**Supplementary Information:**

The online version contains supplementary material available at 10.1186/s13071-025-07015-3.

## Background

*Bolbosoma* Porta, 1908, is a genus of acanthocephalans belonging to the family Polymorphidae Meyer, 1931, which comprises a total of 12 species [1]. *Bolbosoma* species have complex life cycles involving zooplanktonic crustaceans as intermediate hosts and marine mammals (mainly cetaceans) as definitive hosts, and some species are known to include paratenic or transport hosts (mostly teleosts) to facilitate access to appropriate definitive hosts [2–4]. According to host–parasite records (see [5]), seven *Bolbosoma* species seem to occur mainly in mysticetes, i.e., *B. australis* Skrjabin, 1972; *B. balaenae* (Gmelin, 1790); *B. brevicolle* (Malm, 1867); *B. hamiltoni* Baylis, 1929; *B. nipponicum* Yamaguti, 1939; *B. tuberculata* Skrjabin, 1970; and *B. turbinella* (Diesing, 1851), and two in odontocetes, i.e., *B. capitatum* (von Linstow, 1880) and *B. vasculosum* (Rudolphi, 1819). However, most records do not seem to provide quantitative data on infections, nor do they clarify whether the specimens found were sexually mature. Furthermore, three *Bolbosoma* species have been described on the basis of juvenile specimens from fish, namely, *B. caenoforme* (Heitz, 1919), *B. heteracanthe* (Heitz, 1920), and *B. scomberomori* Wang, 1980, with records in other marine vertebrates being apparently scarce [6, 7].

Determining which *Bolbosoma* spp. infections are accidental (i.e., implicate non-hosts), which involve true but atypical hosts (i.e., “straggler” parasites, sensu Rivera-Parra et al. [8]), and which involve true, commonly parasitized hosts is fundamental to understanding patterns of specificity and to accurately trace the history of host–parasite associations [9]. For instance, *B. bobrovoi* Krotov and Delyamure, 1952, was described and subsequently reported in pinnipeds [10–12], but apparently no adult worms have been found in any pinniped species (Additional file 1: Table S1). This species has since been considered as a junior synonym of *B. nipponicum*, a typical parasite of whales, infecting non-hosts [13, 14]. In this context, the case of *B. vasculosum* is particularly intriguing. This species was originally described by Rudolphi (1819) (as *Echinorhynchus vasculosus*) and later reassigned to *Bolbosoma* by Porta (1908), who provided a detailed description based on putative juvenile specimens collected from several fish species and from the short-beaked common dolphin (*Delphinus delphis* [Linnaeus, 1758]). Subsequently, *B. vasculosum* has been reported as juvenile in diverse odontocetes (e.g., Costa et al. [2], Fernández et al. [16], García-Gallego et al. [17], and Van Cleave [18]). On the basis of an analysis of *cox1* nucleotide distances, García-Gallego et al. [17] suggested that at least the immature specimens of *B. vasculosum* found in Mediterranean striped dolphins (*Stenella coeruleoalba* [Meyen, 1833]) could actually represent immature forms of *B. capitatum*. In contrast, two recent surveys found putative adults of *B. vasculosum* in bottlenose dolphins (*Tursiops truncatus* [Montagu, 1821]) and South African fur seals (*Arctocephalus pusillus* [Schreber, 1775]) (see Halajian et al. [19] and Terracciano et al. [20]). García-Gallego et al. [17] highlighted the need for a taxonomic re-analysis of the concept of *B. vasculosum*, including more comprehensive morphological and molecular analyses.

Interestingly, species of *Bolbosoma* are the only acanthocephalans which, along with those of *Corynosoma* Lühe, 1904, are associated with marine mammals [5, 9]. It has been suggested that the polymorphid ancestors of these two genera infected aquatic birds and colonized marine mammals via shared prey, with a subsequent diversification of *Corynosoma* spp. in pinnipeds and *Bolbosoma* spp. in cetaceans [21, 22]. However, the available phylogenetic information for *Bolbosoma* spp. is limited, and their relationships with allied genera of the Polymorphidae are conflicting. In the first comprehensive phylogenetic analysis of this family, García-Varela et al. [21] included data from an unidentified species of *Bolbosoma* and *B. turbinella*. Although both taxa grouped together, they were interspersed among six species of *Corynosoma* in a well-supported clade, which was sister to *Andracantha gravida* (Alegret, 1941), a parasite of cormorants. Subsequent phylogenetic analyses using a higher number of species from these three genera also recovered *Andracantha* + *Corynosoma* + *Bolbosoma* in a well-supported clade, although the monophyly of each genus and/or their relationships are contentious, with rather varied topologies and degrees of statistical support (see García-Varela et al. [23], Gregori et al. [3], Hernández-Orts et al. [24], Lisitsyna et al. [25], Presswell et al. [26], Ru et al. [27], and Santoro et al. [28]).

In the present study, we investigate the concept of *Bolbosoma* and the relationship of species from this genus with allied taxa using a comprehensive approach. First, we examine all available records of *Bolbosoma* spp. in putative definitive hosts to clarify associations for all species of this genus. This helps define patterns of specificity and sheds light on the taxonomic status of species that were described on the basis of juvenile specimens only. In this respect, we pay particular attention to the validity of *B. vasculosum* using a comprehensive morphological and molecular comparison with *B. capitatum* (see García-Gallego et al. [17]). Second, we characterize the complete mitochondrial genome (hereafter mitogenome) of four species, i.e., *B. balaenae*, *B. turbinella*, *B. capitatum*, and *B. vasculosum*. Mitogenomes have provided significant insights into the understanding of the phylogenetic relationships of major groups of acanthocephalans [29–32], but so far have only been described for one *Bolbosoma* species (i.e., *B. nipponicum*) [30]. We reconstruct the phylogenetic relationships of *Bolbosoma* species within the family Polymorphidae, increasing the taxonomic coverage and using mitogenomes when possible. Overall, these analyses will provide a better understanding of the origin and evolution of this group of parasites.

## Methods

### *Host records of *Bolbosoma* spp.*

A thorough bibliographic review of all records on *Bolbosoma* spp. was conducted. The basic source was an unpublished bibliography on acanthocephalans compiled by Dunagan T.T. and Miller D.M. up to the year 2009, which contains over 11,000 references and includes the names of the acanthocephalan species dealt with in each study (available upon request). Other sources used were Amin [1], Delyamure [33], Fraija-Fernández et al. [5], Meyer [34], Petrochenko [35], and Yamaguti [36], as well as references therein, in addition to two databases, namely, the Host–Parasite Database of the Natural History Museum, London, and the Global Biotic Interactions database. Google Scholar was also used to search for primary references from 2009 to the present. The search string used was the complete name of each *Bolbosoma* species, i.e., *B. australis*, *B. balaenae*, *B. brevicolle*, *B. caenoforme*, *B. capitatum*, *B. hamiltoni*, *B. heteracanthe*, *B. nipponicum*, *B. scomberomori*, *B. tuberculata*, *B. turbinella*, and *B. vasculosum*, as well as all the synonyms as indicated by Amin [1] and WoRMS [37]. From each reference we recorded the name of the host species and, when available, information on whether adult worms were present in the sample. Records of *Bolbosoma* spp. in fish were not included in the compilation because fish act as paratenic hosts and only harbor juvenile forms.

### *Collection of *Bolbosoma* specimens*

Specimens of *Bolbosoma* were obtained from cetaceans stranded along the coasts of Spain, Scotland, and Argentina, and from specimens of black scabbard fish, *Aphanopus carbo* Lowe, 1839 (Trichiuridae), collected by fisheries in Madeira, Portugal (Table 1). Permission to collect stranded animals was given by the wildlife services of the regional governments, which are the official institutions in charge of managing and protecting wildlife. During necropsies, intestines were examined in situ for parasites or removed and stored at −20 °C. After thawing, intestines were measured and divided into 20 or 30 sections of equal length. Each section was opened and washed over a 0.02 mm sieve using tap water. The solid content was collected in a Petri dish and examined under a stereomicroscope, and the intestinal wall was thoroughly examined for attached helminths. In the case of fish, specimens were dissected and the visceral cavity was examined under a stereomicroscope. When found, acanthocephalans were left in tap water for 24 h at 4 °C to allow the proboscis to evert, then washed in saline (9 g/L) and preserved in 70% ethanol for morphological analyses or > 95% ethanol for molecular analyses. For identification purposes, acanthocephalans were cleared in lactophenol and diagnostic features were examined following Petrochenko [35], Amin and Margolis [38], and Costa et al. [2]. All *Bolbosoma* specimens were adults based on the presence of eggs in females and sperm in the seminal vesicle of males, except for those found in black scabbard fish and striped dolphins, which were immature worms. Voucher specimens were deposited at the Marine Zoology Unit, Cavanilles Institute of Biodiversity and Evolutionary Biology, University of Valencia, and the Parasitic Worms Collection of the Natural History Museum, under the following registration numbers: *B. balaenae*: MZU FD6-13622, MZU FE1-13647; *B. capitatum*: MZU FE2-13652, MZU FE2-13653; *B. turbinella*: NHMUK 2019.5.5.229-237; *B. vasculosum*: MZU FE1-13631, MZU FE1-13632, MZU FE1-13640, MZU FF2-13837.

**Table 1:** Data on host, locality, and GenBank accession numbers for mitochondrial genomes and/or *cox1*, SSU, and LSU sequences of acanthocephalans used for molecular analyses. Sequences marked with an asterisk were obtained in this study

Acanthocephala species	Host species	Locality	GenBank accession numbers				Tree1	Tree2
			Mitogenome	SSU	LSU	*cox1*		
*Andracantha gravida*	*Phalacrocorax auritus*	Yucatan, Mexico (NA)		EU267802	EU267814	EU267822	x	
*Andracantha sigma*	*Eudyptula minor*	New Zealand (SP)			MF401624	MF527034	x	
*Andracantha leucocarboi*	*Leucocarbo chalconotus*	New Zealand (SP)			MF401623	MF527025	x	
*Andracantha phalacrocoracis*	*Osmerus dentex*	Hokkaido, Japan (NP)			LC461972	LC465354	x	
*Arhythmorhynchus frassoni*	*Eudocimus albus*	Sinaloa, Mexico		JX442165	JX442177		x	
	*Uca spinicarpa*	Yucatan, Mexico				EU189484	x	
*Bolbosoma balaenae*	*Stenella coeruleoalba*	WM	MZ357084*	MZ358109*	MZ366745*		x	x
*Bolbosoma balaenae*	*Nyctiphanes couchii*	NEA		JQ040306		JQ061132	x	
*Bolbosoma balaenae*	*Balaenoptera physalus*	Capri Island (MS)		MZ047218	MZ047238	MZ047279	x	
*Bolbosoma balaenae*	*Balaenoptera physalus*	Capri Island (MS)		MZ047227	MZ047237	MZ047278	x	
*Bolbosoma balaenae*	*Balaenoptera physalus*	Capri Island (MS)		MZ047220	MZ047240	MZ047281	x	
*Bolbosoma balaenae*	*Balaenoptera physalus*	Capri Island (MS)		MZ047224	MZ047234	MZ047275	x	
*Bolbosoma balaenae*	*Balaenoptera physalus*	Capri Island (MS)		MZ047219	MZ047239	MZ047280	x	
*Bolbosoma balaenae*	*Balaenoptera physalus*	Capri Island (MS)		MZ047226	MZ047236	MZ047277	x	
*Bolbosoma balaenae*	*Balaenoptera physalus*	Capri Island (MS)		MZ047225	MZ047235	MZ047276	x	
*Bolbosoma balaenae*	*Balaenoptera physalus*	Capri Island (MS)		MZ047223	MZ047233	MZ047274	x	
*Bolbosoma balaenae*	*Balaenoptera physalus*	Capri Island (MS)		MZ047222	MZ047232	MZ047273	x	
*Bolbosoma balaenae*	*Balaenoptera physalus*	Capri Island (MS)		MZ047221	MZ047231	MZ047272	x	
*Bolbosoma balaenae*	*Stenella coeruleoalba*	WM		**PX273921**	**PX274201**	OR601515	x	
*Bolbosoma caenoforme*	*Salvelinus malma*	Taui Gulf (NP)		KF156879		KF156891	x	
*Bolbosoma capitatum*	*Stenella coeruleoalba*	WM	MZ357085*	MZ358110*	MZ366746*		x	x
*Bolbosoma capitatum*	*Globicephala melas*	WM				OR601511	x	
*Bolbosoma capitatum*	*Pseudorca crassidens*	Argentina (SA)		**PX273922**	**PX274202**		x	
*Bolbosoma capitatum*	*Pseudorca crassidens*	Argentina (SA)				**PX278695**	x	
*Bolbosoma nipponicum*	*Callorhinus ursinus*	St. Paul Island (NP)		ON358429	ON359851	ON359908	x	
*Bolbosoma nipponicum*	*Callorhinus ursinus*	St. Paul Island (NP)		ON361558	ON359856	ON359909	x	
*Bolbosoma nipponicum*	*Callorhinus ursinus*	St. Paul Island (NP)	NC085227					x
*Bolbosoma* sp.	*Callorhinus ursinus*	St. Paul Island (NP)		JX442167	JX442179	JX442190	x	
*Bolbosoma turbinella*	*Eschrichtius robustus*	Monterey Bay, California (NP)		JX442166	JX442178	JX442189	x	
*Bolbosoma turbinella*	*Balaenoptera borealis*	Scotland (NA)	MZ357086*	MZ358111*	MZ366747*		x	x
*Bolbosoma vasculosum*	*Stenella coeruleoalba*	WM	MZ357087*	MZ358112*	MZ366748*		x	x
*Bolbosoma vasculosum*	*Thunnus obesus*	Taiwan (NP)		OR625658		OR625532	x	
*Bolbosoma vasculosum*	*Aphanopus carbo*	Madeira (NA)		**PX273923**	**PX274203**	**PX278696**	x	
*Bolbosoma vasculosum*	*Aphanopus carbo*	Madeira (NA)		**PX273924**	**PX274204**	**PX278697**	x	
*Bolbosoma vasculosum*	*Aphanopus carbo*	Madeira (NA)		**PX273925**	**PX274205**	**PX278698**	x	
*Bolbosoma vasculosum*	*Stenella coeruleoalba*	Cantabrian Sea (NA)		**PX273926**	**PX274206**	**PX278699**	x	
*Bolbosoma vasculosum*	*Stenella coeruleoalba*	Cantabrian Sea (NA)		**PX273927**	**PX274207**	**PX278700**	x	
*Bolbosoma vasculosum*	*Stenella coeruleoalba*	WM		**PX273928**	**PX274208**	OR601513	x	
*Bolbosoma vasculosum*	*Stenella coeruleoalba*	WM		**PX273929**	**PX274209**	OR601514	x	
*Corynosoma australe*	*Phocarctos hookeri*	New Zealand		JX442168	JX442180	JX442191	x	
*Corynosoma enhydri*	*Enhydra lutris*	Monterey Bay, California (NP)		AF001837	AY829107	DQ089719	x	
*Corynosoma magdaleni*	*Phoca hispida saimensis*	Lake Saimaa, Finland		EU267803	EU267815	EF467872	x	
*Corynosoma obtuscens*	*Callorhinus ursinus*	St. Paul Island (NP)		JX442169	JX442181	JX442192	x	
*Corynosoma strumosum*	*Phoca vitulina*	Monterey Bay, California (NP)		EU267804	EU267816	EF467870	x	
*Corynosoma validum*	*Callorhinus ursinus*	St. Paul Island (NP)		JX442170	JX442182	JX442193	x	
*Corynosoma villosum*	*Hippoglossus stenolepis*	St. Paul Island (NP)	NC085226					x
*Hexaglandula corynosoma*	*Nyctanassa violacea*	Veracruz, Mexico		EU267808	EU267817	EF467869	x	
*Ibirhynchus dimorpha*	*Eudocimus albus*	Veracruz, Mexico		GQ981436	GQ981437	GQ981438	x	
*Polymorphus brevis*	*Nycticorax nycticorax*	Michoacan, Mexico		AF064812	AY829105	DQ089717	x	
*Polymorphus minutus*	*Anas platyrhynchos*	Záhlinice, Czech Republic	MN646175				x	x
	*Gammarus pulex*	Dijon, France		EU267806	EU267819		x	x
*Profilicollis bullocki*	*Emerita analoga*	Caleta Lenga, Chile (SP)		JX442174	JX442186	JX442197	x	
*Pseudocorynosoma anatarium*	*Bucephala albeola*	Durango, Mexico		EU267801	EU267813	EU267821	x	
*Pseudocorynosoma constrictum*	*Anas clypeata*	Estado de Mexico, Mexico		EU267800	EU267812	EU267820	x	
*Southwellina hispida*	*Ardea cinerea*	Korea	KJ869251				x	x
	*Epinephelus coioides*	Java, Indonesia		JX014228			x	x
	ND	Baltic Sea, Finland			EU267810		x	x
*Centrorhynchus aluconis*	*Strix aluco*	Czech Republic	KT592357				x	x
	*Strix aluco*	ND		MN057695	MN057699		x	x
*Pomphorhynchus bulbocolli*	ND	ND	NC060483				x	x
	*Onchorhynchus mykiss*	ND		AF001841			x	x
	ND	ND			AY829096		x	x

NA, North Atlantic; SP, South Pacific; NP, North Pacific; WM, Western Mediterranean; NEA, North East Atlantic; MS, Mediterranean Sea; SA, South Atlantic; ND, not determined; SSU, small subunit; LSU, large subunit; Tree1, *cox1* + SSU + LSU tree; Tree2, mtgenome + SSU + LSU tree

### *Morphological analysis of* Bolbosoma vasculosum

Fifteen specimens of *B. vasculosum* collected from the intestines of striped dolphins stranded along the Mediterranean coast of Spain were described. After being cleared in lactophenol, these specimens were drawn using a Nikon Optiphot-2 light microscope (Nikon Instruments, Tokyo, Japan) connected to a drawing tube, and photographed using a Leica Z16 APO Zoom System equipped with a CF500 camera and LAS 4.12 (Leica^®^). Measurements were taken using the software ImageJ v.1.8.0 [39] and were then compared with previous descriptions of *B. vasculosum* [2, 18, 25, 34, 40] and *B. capitatum* [38]. Measurements are in mm unless otherwise stated and are expressed as the range followed by the mean (in parentheses). Width measurements refer to maximum width. Body (= trunk) length does not include neck or proboscis. Specimens of *B. vasculosum* from *A. carbo* were not described in this work since specimens from the same host and location were morphologically described by Costa et al. (2000) [2], but were used for scanning electron microscopy and molecular analyses. Two worms, one from a striped dolphin and another from a black scabbard fish, were dehydrated through an ethanol series (70–100%), critical-point dried, coated with a gold–palladium alloy to a thickness of 250 nm, and viewed and photographed with a Hitachi 4100 FE scanning electron microscope (SEM) (MONOCOMP Instrumentación SA, Madrid, Spain) operating at 20 kV. A specimen of *B. capitatum* collected from a false killer whale, *Pseudorca crassidens* (Owen, 1846) stranded in Argentina, was also photographed using a SEM (JEOL JSM 7401-F) for comparison. Type material (syntypes) of *B. vasculosum* from Atlantic pomfret, *Brama brama* (Bonnaterre, 1788) (nec *Sparus rajus*) and *B. capitatum* from false killer whale deposited at the Museum für Naturkunde, Berlin, Germany (registration numbers 1260-E and 4409-E, respectively), was also examined.

### Molecular analysis

#### DNA extraction, Illumina library preparation, read assembly, and gene annotation

Total genomic DNA was extracted from single individuals using the DNeasy Blood and Tissue (Qiagen) extraction kit or the Monarch^®^ DNA Purification Kit. DNA concentration and quality were measured using a Qubit 2.0 Fluorometer (Life Technologies) and a NanoDrop™ One (Thermo Scientific). Indexed libraries were prepared for four *Bolbosoma* specimens, corresponding to *B. vasculosum*, *B. capitatum*, *B. balaenae*, and *B. turbinella*, using the Nextera DNA Flex Library Prep Kit (Illumina) with 5 ng of input DNA. Quality of Libraries was assessed by quantification and verification of fragment size distribution using a 2200 TapeStation System (Agilent). Libraries from *B. vasculosum* and *B. capitatum* samples were sequenced on an Illumina NextSeq, and those from *B. balaenae* and *B. turbinella* on an Illumina MiSeq.

Initial sample demultiplex and adapter removal was performed using MiSeq Reporter Software (MSR) version 2.6. Subsequently, all Illumina reads were trimmed in Geneious Prime 2019.2.1 using a threshold of > 10% error per base and assembled using SPAdes [41] with the following k-mer sizes: 33, 55, 77, 99, and 127. In Geneious, local BLAST searches of assembled contigs were conducted against a manually curated database of published acanthocephalan mitogenomes and nuclear rDNA sequences. To check assembly coverage, the contigs with the longest mitochondrial (mt) and nuclear rDNA matches were used as seed references for 25 iterative mappings of trimmed reads in Geneious, allowing 3–5% mismatches per read, 14–18-word length, and 1–3% gaps per read. Circularity of mitogenomes was confirmed by aligning ~500 bp from both the start and end of the sequence. To circularize mitogenomes, duplicated data were deleted from one of the two ends.

Mitochondrial gene boundaries were annotated using MITOS [42] using the invertebrate mt translation code (NCBI Table 5). Annotations were manually refined in light of alignments with published acanthocephalan mitogenome sequences. ARWEN [43] was used to identify transfer RNAs (tRNAs) not found by MITOS. Secondary structures of all tRNAs were drawn using mitochondrial-tRNA-Draw (Youngblood and Masta, unpublished) for confirmation of sequences. Gene boundaries of *lsr*- and *ssrDNA* were determined using RNAmmer [44] and annotation was compared with available *lsr*- and *ssrDNA* acanthocephalan sequences.

#### Mitogenome nucleotide diversity (π) inferred by sliding window analysis

Sliding window analyses of nucleotide diversity (number of nucleotide differences per site, π) were performed to explore: (1) the interspecific genetic variability among the mitogenomes of species of Polymorphidae used in this study (i.e., *B. balaenae*, *B. capitatum*, *B. turbinella*, *B. vasculosum*, *Southwellina hispida* (Van Cleave, 1925), and *Polymorphus minutus* (Zeder, 1800); (2) the intraspecific mitogenome genetic variability for the four species of *Bolbosoma* spp.; and (3) variability between representatives of *B. vasculosum* and *B. capitatum*. Nucleotide diversity analyses were conducted in DNAsp version 6 [45] on concatenated alignments of the protein-coding and rRNA genes using a window size of 200 bp and a step size of 10 bp. Gaps in sequence alignments were included in the analysis. Values of π were plotted against the mid points from each sliding window. Pairwise comparisons on nucleotides and translated amino acids were conducted in Geneious.

#### PCR amplification and sequencing of *cox1*, 18S, and 28S regions

Three different genetic markers were partially amplified through PCR, i.e., ~680 bp from the mitochondrial cytochrome *c* oxidase subunit I (*cox1*) region using forward primer LCO1490 5′-GGTCAACAAATCATAAAGATATTGG-3′ and reverse primer HC02198 5′-TAAACTTCAGGGTGACCAAAAAATCA-3′ [46] or forward primer Cory-CO1/F 5′-TGCTTCGTTGGTTTATGTCTTTGA-3′ and reverse primer Cory-CO1/R 5′-CATACTTAACACATAATGAAAATG-3′ [47]; ~1560 bp of the nuclear small subunit (*ssrDNA*) region using forward primer Worm A 5′-GCGAATGGCTCATTAAATCAG-3′ and reverse primer Worm B 5′-CTTGTTACGACTTTTACTTCC-3′ or forward primer 18SA 5′ CCGAATTCGTCGACAACCTGGTTGATCCTGCCAGT 3′ and reverse primer 18SB 5’ CCAGCTTGATCCTTCTGCAGGTTCACCTAC 3′ [48]; and two overlapping fragments of ~1400 and ~1500 bp each of the nuclear large subunit (*lsrDNA*) region using, for the first amplicon (LSU 1), forward primers 5′-CAAGTACCGTGAGGGAAAGTTGC-3′ and reverse primer 5′-CTTCTCCAAC(T/G)TCAGTCTTCAA-3′, and for the second amplicon (LSU 2) forward primer 5′-CTAAGGAGTGTGTAACAACTCACC-3′ and reverse primer 5′-CTTCGCAATGATAGGAAGAGCC-3′ [22]. PCR amplification reactions for each genetic marker were performed in a total volume of 20 μL, including 1.6 μL of both forward and reverse primers (5 μM), 2 μL of template DNA, 10 μL of MyFi™ DNA Polymerase (BioLine, Meridian Life Science Inc., Taunton, MA, USA), and 4.8 μL of PCR water. Cycling conditions for each primer pair were: LCO1490–HC02198: 94 °C for 5 min for an initial denaturation, then 38 cycles of 94 °C for 45 s, 48 °C for 45 s, and 72 °C for 80 s, followed by a final extension at 72 °C for 7 min; Cory-CO1/F-Cory-CO1/R: 95 °C for 3 min, 35 cycles of 94 °C for 1 min, 54 °C for 1 min, and 72 °C for 1 min, followed by 72 °C for 7 min; Worm A–Worm B: 94 °C for 3 min, 40 cycles of 94 °C for 30 s, 56 °C for 30 s, and 72 °C for 2 min, followed by 72 °C for 7 min; 18SA–18SB: 94 °C for 5 min, 30 cycles of 94 °C for 1 min, 50 °C for 1 min, and 72 °C for 1 min, followed by 72 °C for 7 min; and LSU 1 and LSU 2: 94 °C for 3 min, 35 cycles of 94° C for 1 min, 54 °C for 1 min, and 72 °C for 1 min, followed by 72 °C for 7 min. Positive and negative (no template DNA) controls were included in each PCR reaction. PCR products were purified using the Nucleospin^®^ PCR and Gel Purification Clean-up kit (Machery-Nagel, Düren, Germany). Purified amplicons were sent to Macrogen Europe (Amsterdam, the Netherlands) for Sanger sequencing with the same primers used in the PCR amplifications and an additional internal primer for the 18S region: 1270R 5′-CCGTCAATTCCTTTAAGT-3′ [48]. Nucleotide sequences from both strands were used to assemble consensus sequences using Geneious R7 (https://www.geneious.com). The identity of the assembled sequences was verified using the NCBI Basic Local Alignment Search Tool (BLAST) [49].

#### *Cox1* pairwise nucleotide distances of *Bolbosoma* spp.

Mitochondrial *cox1* sequences of *Bolbosoma* spp. and its two closest Polymorphidae genera, i.e., *Corynosoma* spp. and *Andracantha* spp., were aligned and trimmed to match the shortest sequence using Geneious R7. The accuracy of the alignment was inspected by checking the amino acid translation, using the invertebrate mitochondrial code (Translation Table 5). The length of the final alignment was 473 bp and the total number of sequences was 42. Pairwise genetic distances were calculated using the maximum composite likelihood model [50] in MEGA v.11 [51].

#### Phylogenetic analysis

Newly obtained mitogenomes and partial mt *cox1*, *ssr*- and *lsrDNA* sequences from the four *Bolbosoma* spp. together with those available from GenBank (see Table 1 for accession numbers) were used for phylogenetic analyses. Outgroup selection, i.e., *Centrorhynchus aluconis* (Müller, 1780) (Centrorhynchidae) and *Pomphorhynchus bulbocolli* (Linkins, 1919) (Pomphorhynchidae) was informed by the tree topology obtained by Gazi et al. (2016) [29]. No tRNA data were included in the phylogenetic analyses. Data were analysed into four datasets: (1) *ssrDNA* + *lsrDNA* + mitogenome (nucleotides), (2) *ssrDNA* + *lsrDNA* + mt *cox1* sequences (nucleotides), (3) *ssrDNA* + *lsrDNA* + mitogenome (mixed matrix with protein-coding genes as amino acids), and (4) *ssrDNA* + *lsrDNA* + mt *cox1* sequences (mixed matrix with protein-coding genes as amino acids). Datasets including mt *cox1* instead of complete mitogenomes were used to allow for the inclusion of a higher number of sequences and species, particularly those for which mitogenomes were not available. Datasets 1 and 3 had 10 terminals, and datasets 2 and 4 had 54 terminals. Unavailable *ssrDNA*, *lsrDNA*, and/or mt *cox1* were treated as missing data. Genes were aligned separately using the online version of MAFFT [52]. Protein-coding gene nucleotide alignments were edited manually, in frame, using Geneious. Sites of uncertain homology in mt rDNA (*rrnS*, *rrnL*) and nuclear rDNA (*ssrDNA*, *lsrDNA*) were excluded using Gblocks [53] allowing less stringent options, i.e., smaller final blocks, gap positions within blocks, and less strict flanking positions. The evolutionary model for each gene was obtained using the Akaike information criterion in MrModelTest2 [54]. Alignments were concatenated in Geneious. In the concatenated alignment each gene was treated as an independent character set. Bayesian inference (BI) analysis was performed using MrBayes version 3.2.6 [55]. Two parallel runs were carried out using the GTR + I + G model of sequence evolution for nucleotide data and using a mixed model parameter for protein-coding data. Chains were Run for 5,000,000 generations and sampled every 100th generation. The burnin was defined as the point at which the average standard deviation of split frequencies was < 0.01. Maximum likelihood (ML) bootstrap values for 100 replicates were obtained using the Genetic Algorithm for Rapid Likelihood Inference (GARLI) [56] using default settings, partitioned parameters, and models of evolution as used for BI previously described. Clades were considered to have high nodal support if BI posterior probability (BI pp) was > 0.90 and bootstrap values > 80%.

## Results

### Host records of *Bolbosoma* species

**Fig. 1:**
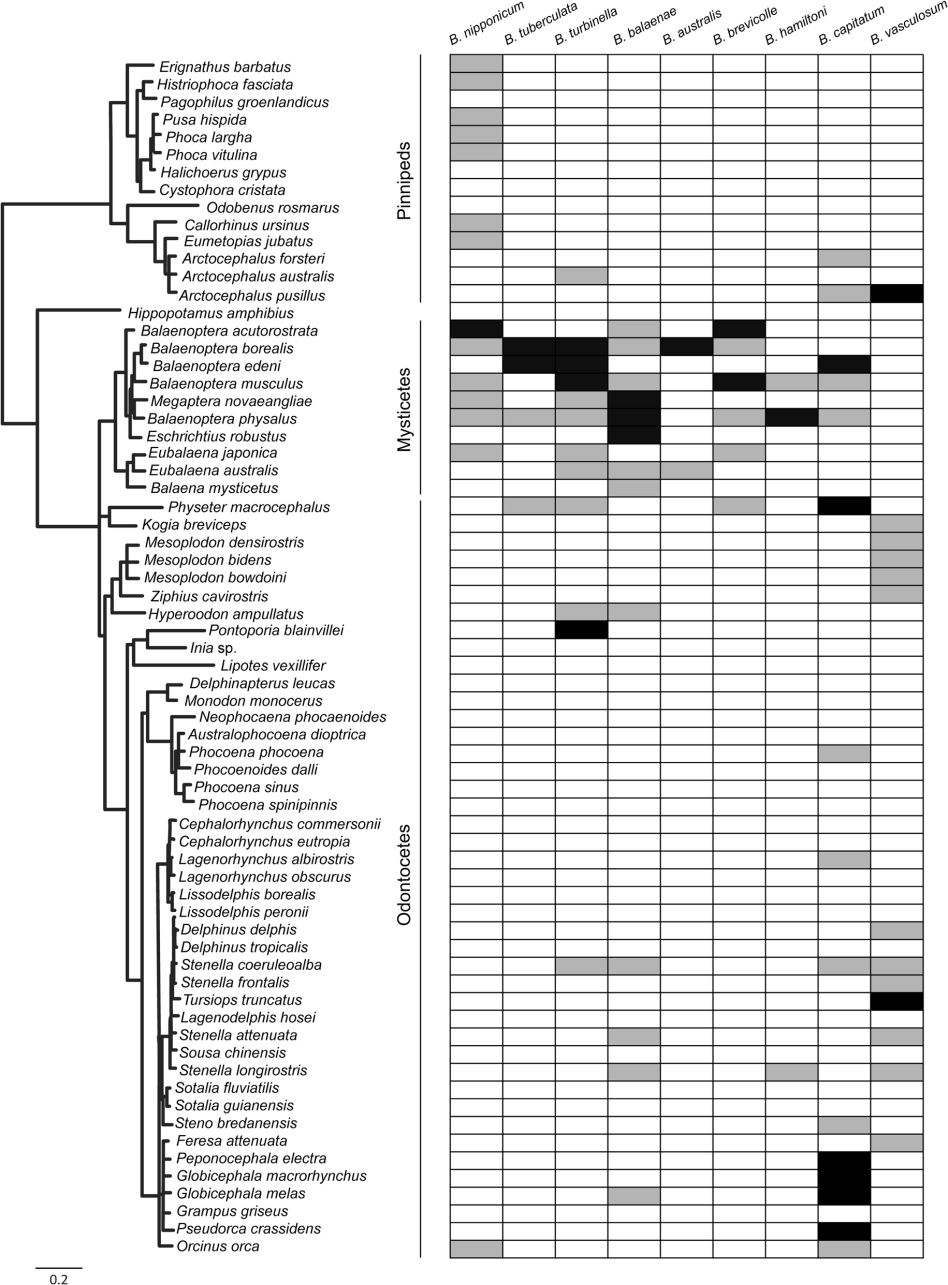
Records of *Bolbosoma* spp. in marine mammals acting as potential final hosts. Records of adult specimens are indicated in black and records of juveniles or specimens for which the maturity stage was not specified are indicated in grey

A total of 375 host–parasite records were compiled for *Bolbosoma* spp., but more than two-thirds of them lacked information on whether or not adult specimens were collected (Additional file 1: Table S1). For the seven *Bolbosoma* species that occur primarily in baleen whales, adult worms were reported in 1–4 host species, mostly from the genus *Balaenoptera* Lacépède, 1804, with records of adult *B. balaenae* also in the humpback whale (*Megaptera novaeangliae* [Borowski, 1781]) and the grey whale (*Eschrichtius robustus* [Lilljeborg, 1861]) (Fig. 1; Additional file 1: Table S1). Juvenile specimens of these species additionally occurred in *Balaena mysticetus* Linnaeus, 1758; *Eubalaena* spp., pinnipeds or odontocetes; and there is a single record of an adult female of *B. turbinella* in the Franciscana dolphin (*Pontoporia blainvillei* [Gervais and d’Orbigny, 1844]) (Fig. 1; Additional file 1: Table S1).

Two *Bolbosoma* species were reported chiefly in odontocetes. Adults of *B. capitatum* were frequently found in four closely delphinid species from the subfamily Globicephalinae, and in the sperm whale (*Physeter macrocephalus* Linnaeus, 1758), with an exceptional record of a mature specimen in the Bryde’s whale (*Balaenoptera edeni* Anderson, 1878); infections in other marine mammals always involved immature worms (Fig. 1; Additional file 1: Table S1). In the case of *B. vasculosum*, nearly all records in potential definitive hosts were in odontocetes, but putative adults were found on a single occasion in common bottlenose dolphins (*T. truncatus*) and in South African fur seals (*A. pusillus*) (Fig. 1; Additional file 1: Table S1).

Juveniles of *B. scomberomori* were detected once in a short-beaked common dolphin (*D. delphis*), and there is one record of *B. caenoforme* in a marine bird, the common murre (*Uria aalge* [Pontoppidan, 1763]), but the maturity stage of the specimens was not specified (Additional file 1: Table S1). We found no records of *B. heteracanthe* in hosts other than fish.

### Morphological description of *B. vasculosum*

**Fig. 2:**
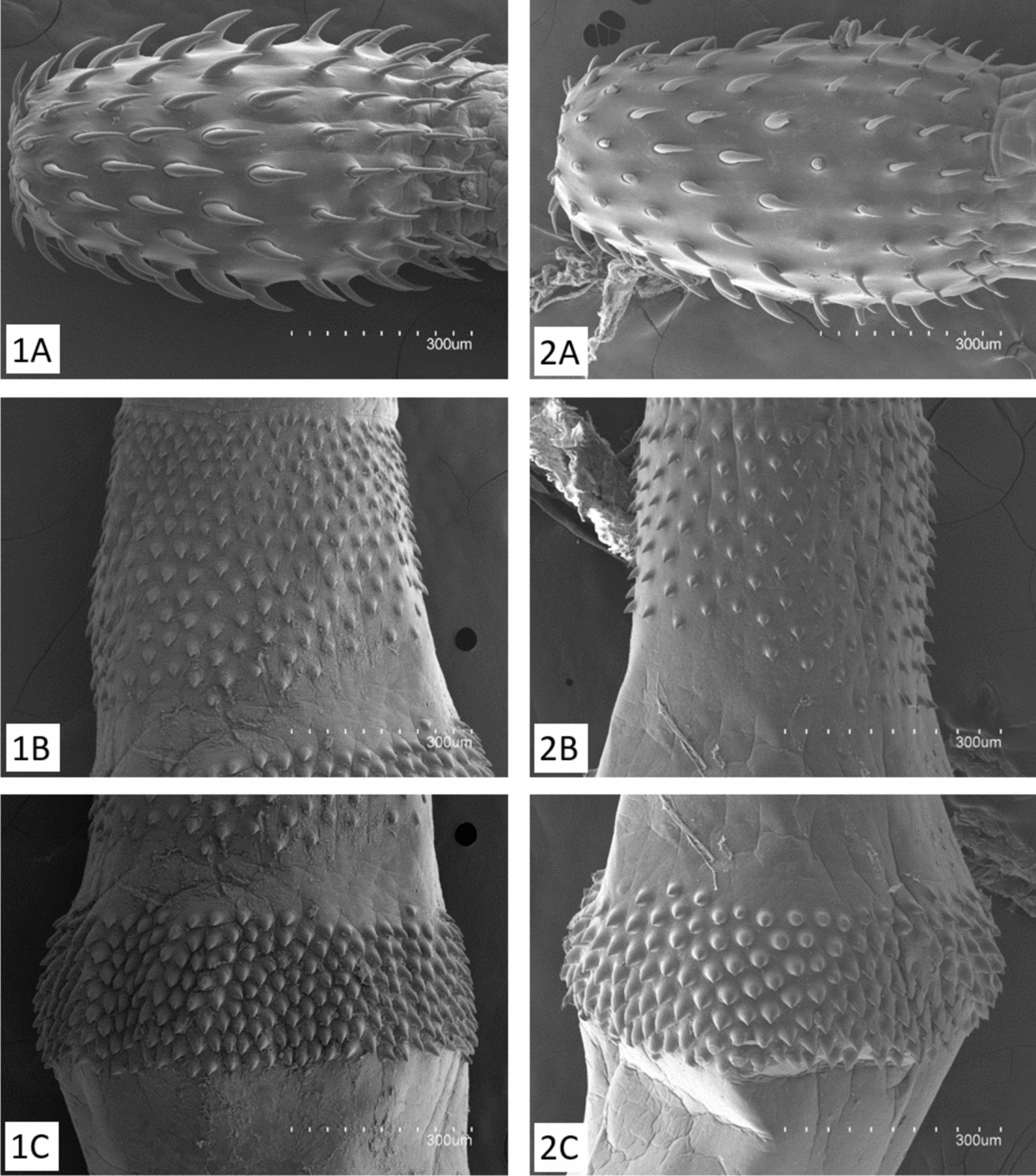
Scanning electron microscopy photographs of *Bolbosoma vasculosum* from (1) *Stenella coeruleoalba* and (2) *Aphanopus carbo*. **A** Proboscis. **B** Anterior field of spines. **C** Posterior field of spines

Specimens collected from both *S. coeruleoalba* and *A. carbo* are closely similar in morphology, with a cylindrical proboscis broadest near its base, having 16–20 longitudinal rows of 7–9 hooks each (Fig. 2). The bulbar region of the foretrunk exhibits two isolated fields of spines separated by a bare area, the anterior field extends more posteriorly on the dorsal side and has 8–14 rings of spines, and the posterior field exhibits a uniform width and has 7–9 rings of spines (Fig. 2).

For the specimens collected from *S. coeruleoalba*, measurements are based on 15 juvenile specimens with no traces of sexual development (number of specimens can be lower depending on the structure measured, see Table 2). Proboscis 605–943 (735) mm long by 370–572 (450) mm wide. Measurements of hook length and width are shown in Table 2. Proboscis receptacle 1.94–2.77 (2.40) mm long by 307–551 (384) mm wide. Neck 503–855 (657) mm long by 432–683 (505) mm wide. Trunk, cylindrical 9.40–22.06 (13.74) mm long by 0.46–1.01 (0.72) mm wide. Anterior bulbar field of spines 287–639 (466) mm long by 553–929 (706) mm wide. Posterior field 223–441 (316) mm long by 0.64–1.29 (0.94) mm wide. The bare area between both fields: 82–254 (149) mm long by 600–950 (816) mm wide, variably free of spines. Lemnisci short, less than half of the proboscis receptacle length, 842–968 (905) mm long.

**Table 2:** Morphometric and meristic data of specimens of *Bolbosoma vasculosum* and *Bolbosoma capitatum* obtained in this and other studies

Species		*B. vasculosum*								*B. capitatum*	
Source		This study	Meyer (1932)	Meyer (1932)	Harada (1925)	Van Cleave (1953)	Costa (2000)	Costa (2000)	Lisitsyna (2023)	Amin and Margolis (1998)	
Sex		–	–	–	Female	–	–	–	Female	Male	Female
Host		*Sc*	Fish	*Dd*	*Tt*	*Mb*	*Ac, Dd*	*Tp*	*To, Tl*	*Pc*	*Pc*
Locality		MS	AO, MS	MS	SJ	NA	NA	NA	NP	NP	NP
*N*		15^a^	?	?	5?	1?	10 and ?	4	2	10	10
Body	L (mm)	13.74 (9.40–22.06) (*n* = 15)	11–12	12–15	8–9.5	12–15	12.25 (11–15)	13.7 (11–15.3)	8.13–8.66	38.62 (33.99–44.88)	49.05 (38.12–68.31)
	W (mm)	0.72 (0.46–1.01) (*n* = 13)	0.75	0.5–0.8	0.4	0.5–0.8	0.55 (0.43–0.66)	0.74 (0.6–0.9)	0.79–0.97	2.33 (1.55–3.66)	2.80 (2.31–3.47)
Proboscis	L (mm)	0.74 (0.61–0.94) (*n* = 7)	*ca*. 1.0	0.76	0.65	0.76–1.00	0.89 (0.72–1.00)	0.77 (0.60–0.90)	0.75–0.68	0.78 (0.72–0.87)	0.87 (0.77–0.95)
	W (mm)	0.45 (0.37–0.57) (*n* = 6)	*ca*. 0.40	0.48	0.40	0.40–0.48	0.49 (0.36–0.60)	0.54 (0.50–0.60)	0.51–0.52	0.52 (0.49–0.55)	0.53 (0.49–0.56)
Proboscis receptacle	L (mm)	2.40 (1.94–2.77) (*n* = 9)	2.29	–	1.5	–	–	–	1.45–1.97	2.95 (2.64–3.37)	3.04 (2.64–3.3)
	W (mm)	0.38 (0.31–0.55) (*n* = 9)	–	–	0.36	–	–	–	0.45–0.49	0.46 (0.40–0.50)	0.50 (0.46–0.59)
Lemnisci	L (mm)	0.91 (0.84–0.97) (*n* = 2)	0.33^b^	–	0.75	3–4.5	–	–	0.89–1.31	3.99 (3.47–4.29)	4.8 (4.03–5.38)
*N* hook rows		16–19 (*n* = 5)	18–20		16–19	18–20	18	–	16	16–18	16–18
Hooks/row		8–9 (*n* = 10)	8–9		8	8–9	8	–	7–9	8–9	8–9
*N* spine rings (anterior)		8–14 (*n* = 7)	–	–	–	–	*ca*. 10	–	8–9	6–13	9–13
*N* spine rings (posterior)		7–9 (*n* = 12)	–	–	–	–	7–8	–	7–8	7–12	8–14
Hook 1	L (µm)	80 (79–81) (*n* = 2)	–	–	86	–	69 (55–80.0)	–	60–80	68 (52–82)	71 (52–78)
	W (µm)	17 (13–22) (*n* = 2)	–	–	20	–	–	–	–	18 (13.26)	19 (13–26)
Hook 2	L (µm)	90 (88–92) (*n* = 2)	–	–	88	–	62^c^ (62–86)	–	75–84	87 (78–96)	87 (78–91)
	W (µm)	22 (13–31) (*n* = 2)	–	–	23	–	–	–	–	23 (20–32)	27 (26–30)
Hook 3	L (µm)	91 (82–97) (*n* = 3)	–	–	100	–	84 (74–92)	–	77–92	95 (91–104)	104 (91–104)
	W (µm)	35 (28–38) (*n* = 3)	–	–	32	–	–	–	–	31 (26–39)	33 (26–45)
Hook 4	L (µm)	95 (88–101) (*n* = 3)	–	–	120	105	85 (74–92)	–	81–94	101 (85–109)	112 (98–130)
	W (µm)	39 (36–44) (*n* = 3)	–	–	40	40–44	–	–	–	41 (26–52)	41 (39–45)
Hook 5	L (µm)	93 (89–98) (*n* = 4)	–	–	78	–	77 (62–92)	–	70–94	108 (91–118)	118 (104–143)
	W (µm)	27 (13–38) (*n* = 5)	–	–	22	–	–	–	–	47 (39–52)	52 (45–58)
Hook 6	L (µm)	88 (66–98) (*n* = 5)	–	–	86	–	75 (62–80)	–	69–83	105 (91–130)	105 (104–111)
	W (µm)	21 (9–30) (*n* = 5)	–	–	20	–	–	–	–	30 (20–39)	51 (45–58)
Hook 7	L (µm)	96 (86–101) (*n* = 5)	–	–	86	–	73 (68–80)	–	75–81	92 (78–104)	95 (78–104)
	W (µm)	18 (15–20) (*n* = 5)	–	–	18	–	–	–	–	23 (20–26)	32 (26–39)
Hook 8	L (µm)	87 (68–104) (*n* = 5)	–	–	76	79	70 (62–80)	–	69–79	94 (85–104)	89 (78–104)
	W (µm)	14 (11–16) (*n* = 5)	–	–	17	16	–	–	–	16 (13–20)	21 (20–26)
Hook 9	L (µm)	84 (*n* = 1)	–	–	–	–	–	–	71	76 (65–91)	86 (65–104)
	W (µm)	9 (*n* = 1)	–	–	–	–	–	–	–	13 (13–16)	17 (13–20)

Data are given as the mean followed by the range when available

*Sc*, *Stenella coeruleoalba*; *Dd*, *Delphinus delphis*; *Tt*, *Thunnus thynnus*; *Mb*, *Mesoplodon bidens*; *Ac*, *Aphanopus carbo*; *Tp*, *Trachurus picturatus*; *To*, *Thunnus obesus*; *Tl*, *Trichiurus lepturus*; *Pc*, *Pseudorca crassidens*; MS, Mediterranean Sea; AO, Atlantic Ocean; SJ, Sea of Japan; NA, North Atlantic; NP, North Pacific

^a^Total number of worms used; *n* can be smaller depending on the structure measured (see number in parentheses for each specific measurement)

^b^Measurement in original description is 33 mm, but most Likely corresponds to 0.33 mm since body size is 11-12 mm

^c^Probably higher because it equates to the minimum value

Meristic and morphometric data of *B. vasculosum* obtained from *S. coeruleoalba* are congruent with previous descriptions of the species based on specimens collected from several fish and dolphins (Table 2). A comparison with the description of adults of *B. capitatum* by Amin and Margolis (1998) [38] demonstrates that all meristic traits, and the morphometry of the proboscis, hooks, and trunk spines, overlap between the two species, except the widths of hooks 5 and 7, which are larger in *B. capitatum* (Table 2).

### Molecular analysis

#### Mitogenome characteristics

Complete mitogenomes and nuclear ribosomal sequences (*ssrDNA* and *lsrDNA*) were obtained via shotgun sequencing from *B. balaenae*, *B. capitatum*, *B. turbinella*, and *B. vasculosum*. A full circular mitogenome (with ~500 bp aligned from both the start and end of the sequence, as described in Section 2.4.1) was only found for *B. balaenae*. However, all mitogenomes recovered full coding regions and featured 12 protein-coding genes (*nad1*–*nad6, nad4L*, *cox1*–*cox3*, *cytb*, and *atp6*) lacking the *atp8* gene, two rRNA genes (*rrnS*, *rrnL*), and 22 tRNA genes. Mitogenome length ranged from 14,199 bp in *B. turbinella* to 14,319 bp in *B. capitatum* (Additional file 2: Table S2). An abbreviated stop codon (T) was found in the *nad6*, *nad3*, and *nad4* genes for all species. One nucleotide overlaps at the 3′ end of *cytb* and the 5′ end of *nad1* was found in all species, and at the 3′ end of *atp6* and the 5′ end of *nad3* in *B. balaenae* and *B. turbinella*. See Additional file 2: Table S2 for specific details on the nucleotide position, codons, and anticodons found for each gene, and Additional files 3–6: Figs. S1–S4 for putative tRNA structures. Nuclear *ssr*- and *lsrDNA* sequences were obtained for the four *Bolbosoma* species. Putative sizes for the nuclear *ssrDNA* and *lsrDNA* were, respectively, 1722 bp and 2220 bp for *B. balaenae*, 1791 bp and 2780 bp for *B. turbinella*, and 1791 bp and 2778 bp for *B. capitatum* and *B. vasculosum*.

#### Mitogenome nucleotide diversity

**Fig. 3:**
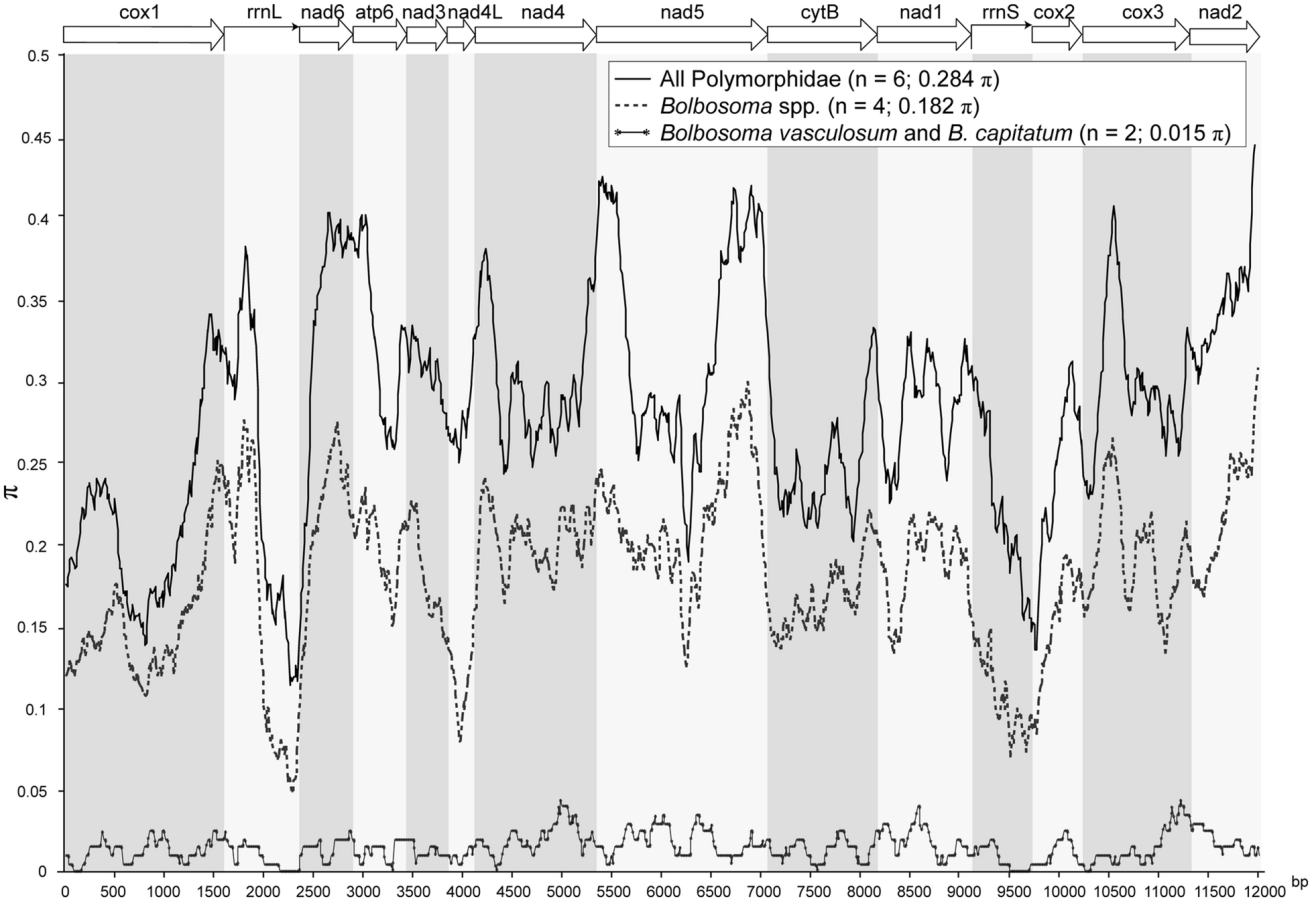
Sliding window analysis of mitochondrial genomes of *Bolbosoma* spp. π, number of nucleotide differences per site; bp, base pair

Among the six sequences of the Polymorphidae a total of 6442 polymorphic sites were found in the protein-coding and rRNA genes, and overall, pairwise nucleotide diversity was 0.284 π (Fig. 3). On average, the highest nucleotide diversity for the Polymorphidae was found within the *nad6* (π = 0.378), *nad2* (π = 0.360), and *nad5* (π = 0.331) genes, whilst the lowest values were within the *cox1* (π = 0.206) and rRNA genes (π = 0.213 in *rrnS*; π = 0.243 in *rrnL*) (Fig. 3). Interspecific variability between the four samples of *Bolbosoma* spp. included a total of 3626 polymorphic sites, an overall pairwise nucleotide diversity of 0.182 π (Fig. 3), and a nucleotide similarity of 81.4% in protein-coding and rRNA genes. Nucleotide diversity within genes between samples ranged from 0.104 π in *rrnS*, 0.119 π in *nad4L*, 0.142 π in *cox1*, 0.239 π in *nad6*, 0.233 π in *nad2*, and 0.216 π in *nad5* (Fig. 3). Specifically, for *B. capitatum* and *B. vasculosum*, there were only 176 polymorphic sites out of the 12,056 sites compared among protein-coding and rRNA genes, as Little as 0.015 π of nucleotide diversity, and 98.5% pairwise nucleotide similarity (Fig. 3).

#### *Cox1* genetic distances

Based on *cox1* sequences, the range of interspecific genetic distances between species of *Bolbosoma* and *Andracantha* was 0.284–0.448, and between *Bolbosoma* and *Corynosoma* 0.258–0.392 (Additional file 7: Table S3). Among *Bolbosoma* spp., distances ranged from 0.002 to 0.274; the closest species were *B. caenoforme* and *B. nipponicum* (0.002), and *B. vasculosum* and *B. capitatum* (0.028), with the minimum genetic distance between any other two *Bolbosoma* spp. being 0.176. At the intraspecific level, genetic distances were as follows: 0.000–0.017 (*B. balaenae*), 0.011–0-015 (*B. capitatum*), 0.004 (*B. nipponicum*), 0.151 (*B. turbinella*), and 0.000–0.026 (*B. vasculosum*).

#### Phylogenetic analyses

**Fig. 4:**
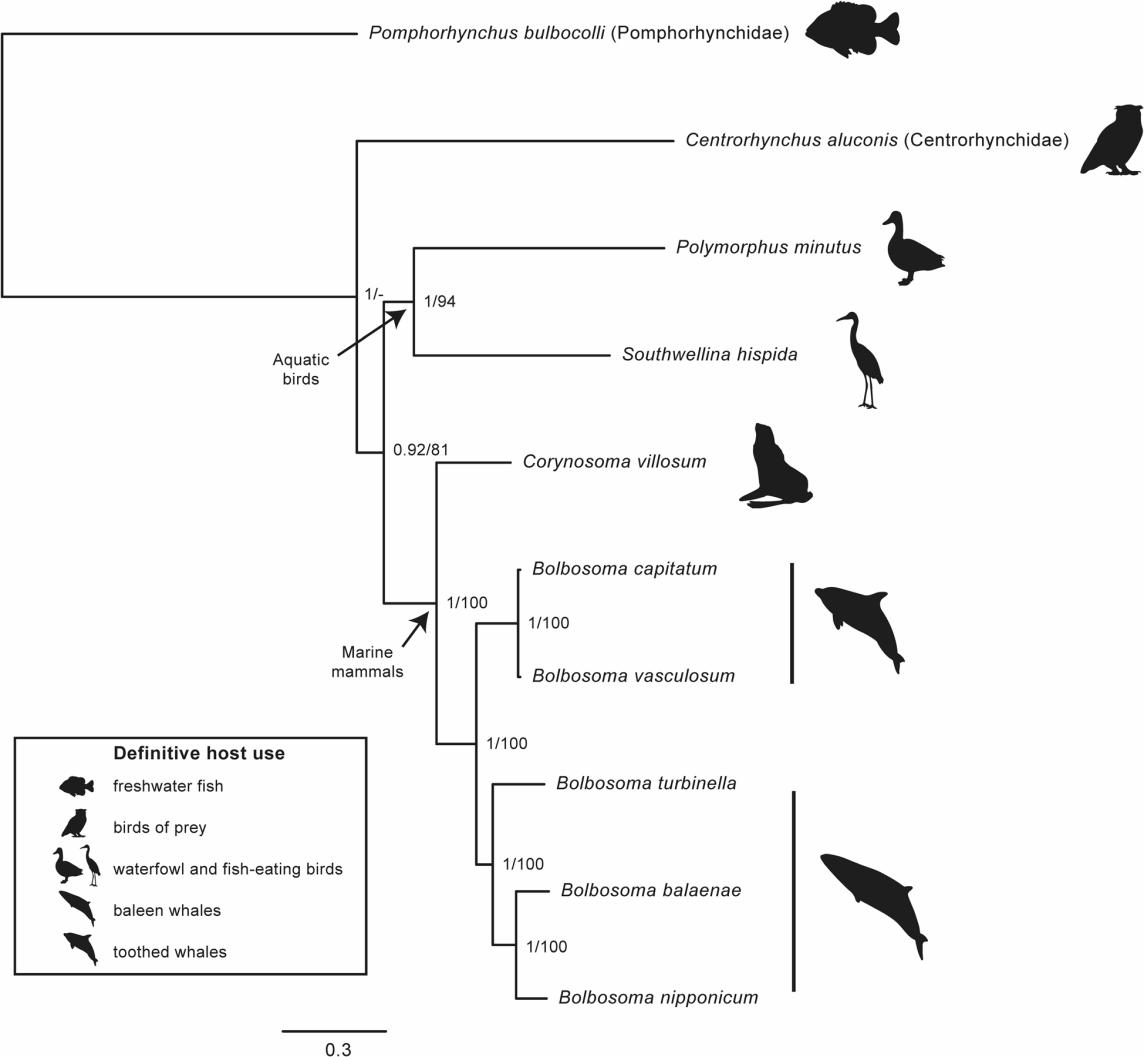
Phylogenetic relationships of *Bolbosoma* spp. and related Acanthocephala based on Bayesian and maximum likelihood analyses of mitogenome (nucleotides) + small subunit ribosomal DNA (ssrDNA) + large subunit ribosomal DNA (lsrDNA) data. *Centrorhynchus aluconis* (Centrorhynchidae) and *Pomphorhynchus bulbocolli* (Pomphorhynchidae) were used as outgroups. Posterior probabilities and maximum likelihood bootstrap support values are given for each node. Scale bar indicates number of substitutions per site. Definitive host use is indicated as in the legend

**Fig. 5:**
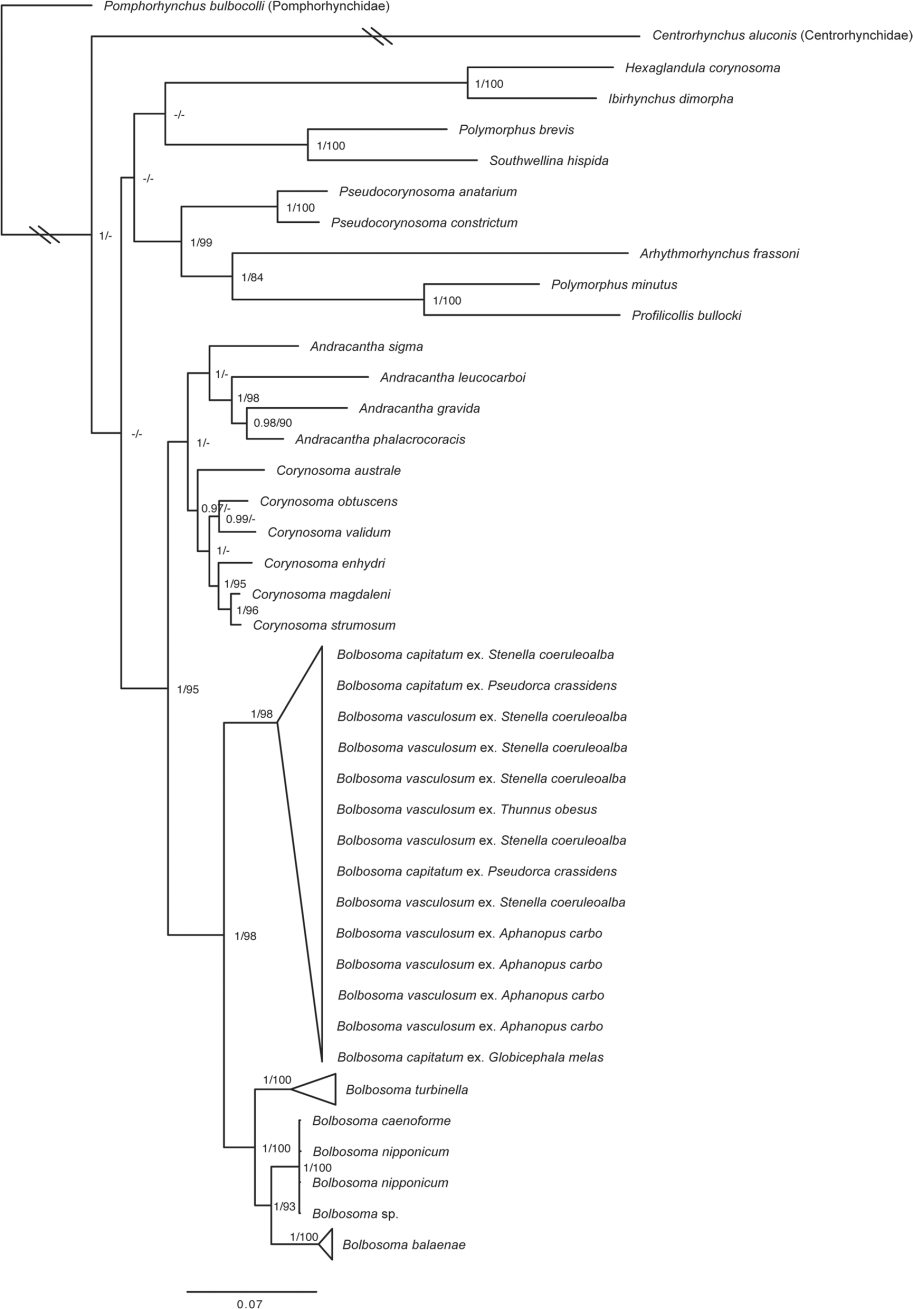
Phylogenetic relationships of *Bolbosoma* spp. and related Acanthocephala based on Bayesian and maximum likelihood analyses of cytochrome *c* oxidase subunit I (*cox1*) (nucleotides) + small subunit ribosomal DNA (ssrDNA) + large subunit ribosomal DNA (lsrDNA) data. *Centrorhynchus aluconis* (Centrorhynchidae) and *Pomphorhynchus bulbocolli* (Pomphorhynchidae) were used as outgroups. Posterior probabilities and maximum likelihood bootstrap support values are given for each node. Posterior probabilities < 0.9 and bootstrap support values < 80% are not shown. Scale bar indicates number of substitutions per site

The *ssrDNA* + *lsrDNA* + mitogenome (nucleotides) (dataset 1) consisted of ten terminals and 17,025 aligned positions after excluding 28 positions in the nuclear rDNA genes, which were ambiguously aligned. The *ssrDNA* + *lsrDNA* + mt *cox1* (nucleotides) (dataset 2) consisted of 54 terminals and 6040 aligned positions, after excluding 780 ambiguously aligned sites in the nuclear rDNA genes. The *ssrDNA* + *lsrDNA* + mitogenome (mixed matrix with protein-coding genes as amino acids) (dataset 3) consisted of ten terminals and 9750 positions, and the *ssrDNA* + *lsrDNA* + mt *cox1* sequences (mixed matrix with protein-coding genes as amino acids) (dataset 4) consisted of 54 terminals and 5009 positions. In all datasets, the selected outgroups *Centrorhynchus aluconis* (Centrorhynchidae) and *Pomphorhynchus bulbocolli* (Pomphorhynchidae) formed separate clades from all Polymorphidae species (Figs. 4 and 5; Additional files 8 and 9: Figs. S5 and S6). Within the Polymorphidae, tree topologies and nodal support values were also consistent across all datasets, except for the interrelationship of *Polymorphus minutus* and *Southwellina hispida* (Figs. 4 and 5; Additional files 8 and 9: Figs. S5 and S6). Among these, *Andracantha* spp., *Corynosoma* spp., and *Bolbosoma* spp. were recovered as a sister clade to the other taxa (Fig. 5; Additional file 9: Fig. S6). All *Bolbosoma* spp. formed a well-supported clade by Bayesian pp and ML bs values in all datasets (pp = 1.00: bs ≥ 98%; Figs. 4 and 5; Additional files 8 and 9: Figs. S5 and S6), and *Andracantha* spp. and *Corynosoma* spp. were recovered as sister clades (Fig. 5; Additional file 9: Fig. S6). Within the clade including *Bolbosoma* spp., all datasets rendered *B. capitatum*, specimens from *P. crassidens*, long-finned pilot whale (*Globicephala melas* [Traill, 1809]) and *S. coeruleoalba*; and *B. vasculosum*, specimens from *A. carbo*, *Thunnus obesus* (Lowe, 1839) (Scombridae), and *S. coeruleoalba*, in a well-supported clade (pp = 1.00; bs ≥ 98%; Figs. 4 and 5; Additional files 8 and 9: Figs. S5 and S6). Among the remaining species, *B turbinella* was recovered as sister to a clade formed by *B. caenoforme* + *B. nipponicum* + *Bolbosoma* sp. and *B. balaenae* (Fig. 5; Additional file 9: Fig. S6).

## Discussion

### Host-parasite associations

Results from the present study reveal that records of *Bolbosoma* spp. in putative definitive hosts, even those recently published, often lack basic information on the degree of sexual maturity of the specimens found, thus we urge authors to always provide it in future parasitological surveys. Based on the available evidence, the validity of three *Bolbosoma* species seems to be problematic because there are no confirmed records of adults. *Bolbosoma heteracanthe* was described from a single juvenile collected from Atlantic salmon (*Salmo salar* L.) in continental Europe [34] and has only been reported once more in fish from the tropical Pacific [57]. The World Register of Marine Species considers this species as a *taxon inquerendum*, as we do here, because the description in Meyer (1932) does not provide enough information to separate *B. heteracanthe* from other *Bolbosoma* spp. The second species, *B. caenoforme*, has frequently been reported as juvenile in fish [58], except for a single record in a marine bird, the common murre (*U. aalge*), in which the maturity stage of the specimens was unclear [6]. Yamaguti [36] hypothesized that *B. caenoforme* may be synonymous with *B. nipponicum*, as did Kita et al. [58], on the basis of genetic and morphological similarities. The present study confirms a small genetic distance between these two species based on *cox1* sequences (0.002), which is within the range of intraspecific variation observed for other *Bolbosoma* species (Additional file 7: Table S3). Finally, *B. scomberomori* was described as juvenile in fish, with just one record in a short-beaked common dolphin (*D. delphis*) in which no gravid females were found [7]. This species is morphologically close to *B. nipponicum* and only subtle differences separate both species [7].

There are seven *Bolbosoma* species that have regularly been found as adults in host species from the mysticete clade that includes *Balaenoptera* spp. + *M. novaeangliae* + *E. robustus*, i.e., the Balaenopteroidea [59]. Pending further evidence, these parasites would be unable to reproduce, or would hardly do so, in balaenids, odontocetes, and pinnipeds, which get infected by the consumption of shared intermediate and/or paratenic hosts. Conversely, *B. capitatum* seems to be associated with globicephaline delphinids around the world [38], often producing heavy infections [60, 61]. Sperm whales also seem to be compatible hosts for this species, but *B. capitatum* seems unable to mature in other marine mammals. In this respect, the finding of adults of *B. capitatum* in Bryde’s whale seems doubtful because Pinto et al. [62] reported a single field of bulbar spines in their specimens, which is typical for some species infecting baleen whales (e.g., *B. turbinella* or *B. nipponicum*), but not for *B. capitatum*, which has two fields [35, 38].

The case of *B. vasculosum* is the most intriguing. Records of juveniles of this species seem to be widespread in odontocetes, but we found only four records of putative adults (Additional file 1: Table S1). Harada [40] reported the presence of eggs in three females collected from Atlantic bluefin tuna (*Thunnus thynnus*), and Williams and Bunkley-Williams [63] reported one adult (not sexed) in king mackerel (*Scomberomorus cavalla*). These records, if valid, would be exceptional since fish typically act as paratenic hosts for species of *Bolbosoma* [2, 58, 64]. However, Terracciano et al. (2020) [20] pictured an adult female of *B. vasculosum* in a common bottlenose dolphin (*T. truncatus*), but the specimen was tiny (0.85 mm long by 0.435 mm wide) and the putative eggs exhibited an unusual shape and were far smaller than any of typical *Bolbosoma* spp. Finally, Halajian et al. [19] reported adults in Cape fur seals (*A. pusillus*), which the authors considered an exceptional host. This material, consisting of three females, was re-examined and although the specimens appeared to have germ cell balls, none of them had eggs, indicating individuals were not mature. The taxonomic status of *B. vasculosum* is discussed in the next section.

### Taxonomic status of *Bolbosoma vasculosum*

The main diagnostic characters used to discriminate among species of *Bolbosoma* are the number of longitudinal rows of spines in the proboscis, the number of hooks per row, and the pattern of trunk spination [18, 35, 38]. These features have been observed to remain unchanged from the cystacanth to the adult stage (see, e.g., Ru et al. [27] for *Bolbosoma* spp.).

All descriptions of specimens identified (or synonymized) as *B. vasculosum* in literature, and the new description provided in the present paper, which are based on cystacanths from fish or juveniles from odontocetes, conform to the description by Porta [15] for this species. When compared with the thorough redescription of adults of *B. capitatum* by Amin and Margolis [38], a close morphological similarity arises. These are the only two *Bolbosoma* species with two separated fields of spines in the foretrunk [34–36], and the number of spine rings in each field overlaps between the two species. This overlap also occurs in the number of longitudinal rows of hooks (16–20 in *B. vasculosum* versus 16–18 in *B. capitatum*), and both species have 8–9 hooks per row (Fig. 2; Additional file 10: Fig. S7). There are, however, morphometric differences between both species that result from specimens of each being in different developmental stages, i.e., cystacanth or juvenile in *B. vasculosum* versus adult in *B. capitatum*. It is noteworthy that the lemnisci are clearly longer in *B. capitatum*, but a similar difference has been observed between juvenile and adult specimens of the same *Bolbosoma* species [27], thus it should not be used as a diagnostic trait when comparing different developmental stages (*contra* Harada [40]). Furthermore, hooks 5 and 7, but not the others, are larger in *B. capitatum* than in *B. vasculosum*. However, this would not be particularly surprising, as in allied polymorphid species, differential growth of holdfast structures have been detected during adult development, and it has been interpreted as an adaptation to a fine-tuned adjustment to attach to the definitive hosts [65].

Overall, we see no morphological ground to justify considering *B. vasculosum* and *B. capitatum* as separate species, and this conclusion is clearly supported by the molecular analyses. In the present study, we expanded the preliminary study by García-Gallego et al. [17] with a great deal of sequences from *B. vasculosum* and *B. capitatum*, and the genetic distances found between all specimens fell within the range of intraspecific variation for other *Bolbosoma* species (Additional file 7: Table S3). Similarly, when comparing the mitogenomes, we found a nucleotide diversity for protein coding and rRNA genes as Little as 0.015 π for *B. vasculosum* and *B. capitatum*, as compared with 0.183 π for all *Bolbosoma* spp. (Fig. 3). Moreover, phylogenetic analyses resulting from the four different datasets used in our study showed a strongly supported clade that included the sequences of *B. vasculosum* interspersed with those of *B. capitatum*.

In summary, we conclude that *B. vasculosum* and *B. capitatum* are conspecific and, since *B. capitatum* is the junior synonym, the species should be named as *B. vasculosum*. Patterns of specificity derived from host records (see the previous section) indicate that *B. vasculosum* is specific to globicephaline odontocetes and sperm whales, with other cetaceans being unsuitable hosts for this species. The record of adults of *B. vasculosum* in Cape fur seals requires further clarification.

### Phylogenetic relationships

The newly sequenced mitogenome, *cox1*, and nuclear ribosomal sequences indicate that species of *Bolbosoma* constitute a highly supported monophyletic group, in accordance with previous research [25, 28]. Furthermore, our study shows, for the first time, a well-supported clade divergence between *B. vasculosum* and *B. capitatum* (now considered conspecific) on one side, and *B. turbinella* + *B. caenoforme* + *B. nipponicum* + *B. balaenae* on the other. This topology conforms to the patterns of specificity noted above and, overall, would suggest the existence of a “toothed-whale clade” and a “baleen-whale clade” for *Bolbosoma* spp. The inclusion of further species specific to mysticetes, i.e., *B. australis*, *B. brevicolle*, and *B. hamiltoni*, will help confirm or reject this hypothesis.

Our phylogenetic analysis also provides support for the monophyly of *Corynosoma* and *Andracantha*, rendering *Bolbosoma* as a sister taxon to these two genera. Although a similar topology was recovered by García-Varela et al. [23] and Gregori et al. [3], most previous studies found *Andracantha* to be sister to the clade formed by *Corynosoma* and *Bolbosoma* [21, 26–28], whereas Lisitsyna et al. [25] recovered *Corynosoma* as sister to *Andracantha* + *Bolbosoma*. It should be emphasized, however, that in the present study: (i) the broadest range of *Bolbosoma* species are included; (ii) the cladogram conforms to the current taxonomy, which recognizes the three genera as valid; and (iii) all nodes received strong statistical support.

The closeness between species of *Corynosoma* and *Andracantha* is also suggested by morphological resemblance [66]; indeed, some species of *Andracantha* were previously classified as members of *Corynosoma* (see Schmidt [67]). Moreover, the available evidence suggests that the intermediate hosts for *Corynosoma* spp. are amphipods [68, 69], which are also presumed to be for *Andracantha* spp. [70]. In contrast, euphausiids are the only intermediate hosts reported for *Bolbosoma* spp. so far [3].

Species of *Bolbosoma*, *Corynosoma*, and *Andracantha* occur worldwide in cetaceans, pinnipeds, and marine birds (especially cormorants), respectively [66, 67, 71], which suggests an ancient colonization of the marine environment by a polymorphid ancestor, with subsequent host-switching events among these phylogenetically unrelated hosts [21]. In the case of *Bolbosoma* spp., the phylogeny presented here points to an association with cetaceans via a host-switching event from aquatic birds. How this may have occurred is an interesting question. On the basis of a parsimony criterion, García-Varela et al. [21] showed that the polymorphid ancestors that colonized marine mammals would use amphipods as intermediate hosts; a plesiomorphic character that seems to have been retained in *Corynosoma* and, presumably, *Andracantha*. Thus, the shift to euphausiids as intermediate hosts in *Bolbosoma* spp. would be a further step that allowed infection of modern baleen whales through the oceanic trophic webs.

A plausible historical reconstruction of this intermediate host switch would require firstly to date the origin of associations between *Bolbosoma* spp. and cetaceans. According to our results, two scenarios are possible. If the association predated the split between odontocetes and mysticetes, the presence of *B. vasculosum* (= *capitatum*) in some odontocetes could be interpreted as relictual. However, if the original association of *Bolbosoma* occurred after the split, the occurrence of *B. vasculosum* in some odontocetes should have resulted from host-switching. To clarify this issue, knowing the identity of the (unknown) intermediate hosts of *B. vasculosum* will be instrumental, particularly if amphipods, and not euphasiids, were involved.

## Conclusions

The results obtained in this study indicate that *Bolbosoma* spp. are specific to cetaceans, while the remaining reported hosts are most likely atypical. The taxonomy and identity of species described on the basis of juvenile specimens (i.e., *B. caenoforme*, *B. heteracanthe*, and *B. scomberomori*) should be revisited, as evidence suggests they correspond to the juvenile forms of other known species. This is illustrated by the case of *B. vasculosum*, which is here synonymized to *B. capitatum*. This case reinforces the idea that *Bolbosoma* spp. exhibit clear specificity patterns, not only to cetaceans as a whole but also to odontocetes (in the case of *B. vasculosum*) or mystsicetes (for the remaining *Bolbosoma* species). The phylogeny obtained provides, for the first time, substantial support for the monophyly of *Andracantha* spp., *Corynosoma* spp., and *Bolbosoma* spp., clarifying the relationships among these three genera. Accordingly, *Bolbosoma* spp. seem to have originated from host-switching events in the marine realm. Such events have been repeatedly reported in cetacean parasites, whose origin most likely stems from adaptations to new hosts during their transition to the marine environment.

## Supplementary Information

Table S1. List of Bolbosoma species and potential definitive hosts, indicating maturity stage of specimens.

Table S2. Mitogenome boundaries of Bolbosoma capitatum, Bolbosoma vasculosum, Bolbosoma balaenae and Bolbosoma turbinella.

Figure S1. Putative secondary structures of the 22 tRNAs identified in the mitochondrial genome of Bolbosoma balaenae.

Figure S2. Putative secondary structures of the 22 tRNAs identified in the mitochondrial genome of Bolbosoma capitatum.

Figure S3. Putative secondary structures of the 22 tRNAs identified in the mitochondrial genome of Bolbosoma turbinella.

Figure S4. Putative secondary structures of the 22 tRNAs identified in the mitochondrial genome of Bolbosoma vasculosum.

Table S3. Pairwise genetic distances matrix based on partial mitochondrial cox1 sequences of Andracantha gravida, Andracantha leucocarboi, Andracantha phalacrocoracis, Andracantha sigma, Bolbosoma balaenae, Bolbosoma caenoforme, Bolbosoma capitatum, Bolbosoma nipponicum, Bolbosoma sp., Bolbosoma turbinella, Bolbosoma vasculosum, Corynosoma australe, Corynosoma enhydri, Corynosoma magdaleni, Corynosoma obtuscens, Corynosoma strumosum and Corynosoma validum.

Figure S5. Phylogenetic relationships of Bolbosoma spp. and related Acanthocephala based on Bayesian and Maximum Likelihood analyses of mitogenome (mixed matrix with protein-coding genes as amino acids) + small subunit ribosomal DNA (ssrDNA) + large subunit ribosomal DNA (lsrDNA) data. Centrorhynchus aluconis (Centrorhynchidae) and Pomphorhynchus bulbocolli (Pomphorhynchidae) were used as outgroups. Posterior probabilities and maximum likelihood bootstrap support values are given for each node. Bootstrap support values < 80% are not shown. Scale bar indicates number of substitutions per site.

Figure S6. Phylogenetic relationships of Bolbosoma spp. and related Acanthocephala based on Bayesian and Maximum Likelihood analyses of cytochrome c oxidase subunit I (cox1) (as amino acids) + small subunit ribosomal DNA (ssrDNA) + large subunit ribosomal DNA (lsrDNA) data. Centrorhynchus aluconis (Centrorhynchidae) and Pomphorhynchus bulbocolli (Pomphorhynchidae) were used as outgroups. Posterior probabilities and maximum likelihood bootstrap support values are given for each node. Posterior probabilities < 0.9 and bootstrap support values < 80% are not shown. Scale bar indicates number of substitutions per site.

Figure S7. Scanning electron microscopy photographs of *Bolbosoma capitatum* from *Pseudorca crassidens*.

## Data Availability

Data supporting the conclusions of this article are included within the article and its additional files. Sequences generated and analyzed in this study are available in the NCBI GenBank database (https://www.ncbi.nlm.nih.gov/genbank/) and can be accessed through the accession numbers provided in the article. Voucher specimens have been deposited at the Marine Zoology Unit, Cavanilles Institute of Biodiversity and Evolutionary Biology, University of Valencia, and the Parasitic Worms Collection of the Natural History Museum under the registration numbers listed in the article. Any additional data are available from the corresponding author upon request.

## References

[1] Amin OM. Classification of the Acanthocephala. Folia Parasitol (Praha). 2013;60:273–305. 24261131 10.14411/fp.2013.031

[2] Costa G, Chubb JC, Veltkamp CJ. Cystacanths of *Bolbosoma vasculosum* in the black scabbard fish *Aphanopus carbo*, oceanic horse mackerel *Trachurus picturatus* and common dolphin *Delphinus delphis* from Madeira. Portugal J Helminthol. 2000;74:113–20. 10881281

[3] Gregori M, Aznar FJ, Abollo E, Roura Á, González ÁF, Pascual S. *Nyctiphanes couchii* as intermediate host for the acanthocephalan *Bolbosoma balaenae* in temperate waters of the NE Atlantic. Dis Aquat Organ. 2012;99:37–47. 22585301 10.3354/dao02457

[4] Kennedy CR. Ecology of the Acanthocephala. New York: Cambridge University Press; 2006. p. 211.

[5] Fraija-Fernández N, Fernández M, Raga JA, Aznar FJ. Helminth diversity of cetaceans: an update. Adv Mar Biol. 2016;1:29–100.

[6] Kihara M. Studies on the Acanthocephala (V). An Acanthocephala of *Uria aalge inornata* Salomonsen of northern ocean. Proc Jpn Parasitol Soc East Jpn Sect. 1959;19: 30 [In Japanese].

[7] Wang PQ. Notes on the Acanthocephala from Fugian. II Acta Zootaxonomica Sin. 1980;2:116–23.

[8] Rivera-Parra JL, Levin II, Johnson KP, Parker PG. Host sympatry and body size influence parasite straggling rate in a highly connected multihost, multiparasite system. Ecol Evol. 2017;7:3724–31. 28616169 10.1002/ece3.2971 PMC5468160

[9] Hernández-Orts JS, Brandão M, Georgieva S, Raga JA, Crespo EA, Luque JL, et al. From mammals back to birds: Host-switch of the acanthocephalan *Corynosoma australe* from pinnipeds to the Magellanic penguin *Spheniscus magellanicus*. PLoS ONE. 2017;12:e0183809. 28981550 10.1371/journal.pone.0183809 PMC5628790

[10] Dailey MD. The distribution and intraspecific variation of helminth parasites in pinnipeds. Rapp Proces-Verbaux Reun Cons Int Pour Explor Mer. 1975;169:338–52.

[11] Krotov AI, Delyamure SL. On the helminth fauna of mammals and birds of the SSSR. Tr Gelmintologicheskoi Lab Akad Nauk SSSR. 1952;6:278–92.

[12] Shults LM. Helminth parasites of the Steller sea lion, *Eumetopias jubatus*, in Alaska. In: Proceedings of the Helminthological Society of Washington. 1986. p. 194–7.

[13] Kuzmina TA, Lisitsyna OI, Lyons ET, Spraker TR, Tolliver SC. Acanthocephalans in northern fur seals (*Callorhinus ursinus*) and a harbor seal (*Phoca vitulina*) on St. Paul Island, Alaska: species, prevalence, and biodiversity in four fur seal subpopulations. Parasitol Res. 2012;11:1049–58. 10.1007/s00436-012-2930-x 22584377

[14] Yurakhno MV, Loboda AP, Stryukov AA, Solov’ov VV. About validity of species *Bolbosoma bobrovi* (Acanthocephala, Polymorphidae). Vestn Zool Suppl. 2005;19:371–373 [In Russian].

[15] Porta A. Gli Acantocefali dei mammiferi. Nota preventiva Arch Parasitol Paris. 1908;12:268–82.

[16] Fernández M, Aznar FJ, Montero FE, Georgiev BB, Raga JA. Gastrointestinal helminths of Cuvier’s beaked whales, *Ziphius cavirostris*, from the western Mediterranean. J Parasitol. 2004;90:418–20. 15165073 10.1645/GE-105R

[17] García-Gallego A, Raga JA, Fraija-Fernández N, Aznar FJ. Temporal and geographical changes in the intestinal helminth fauna of striped dolphins, *Stenella coeruleoalba*, in the western Mediterranean: a long-term analysis (1982–2016). Front Mar Sci. 2023;10:1272353.

[18] Van Cleave HJ. Acanthocephala of North American mammals. Ill Biol Monogr. 1953;23(1/2).

[19] Halajian A, Smales L, Heckmann R, Amakali AM, Tjipute M, Wilhelm MR, et al. *Corynosoma australe* and *Bolbosoma vasculosum* (Polymorphidae: Acanthocephala) from *Arctocephalus pusillus pusillus* (Otariidae) and *Argyrosomus* spp. (Sciaenidae) from the Namibian Coast of Africa. Comp Parasitol. 2020;87:127–34.

[20] Terracciano G, Fichi G, Comentale A, Ricci E, Mancusi C, Perrucci S. Dolphins stranded along the Tuscan coastline (central Italy) of the “Pelagos Sanctuary”: a parasitological investigation. Pathogens. 2020;9:612. 32727040 10.3390/pathogens9080612 PMC7459703

[21] García-Varela M, Pérez-Ponce de León G, Aznar FJ, Nadler SA. Phylogenetic relationship among genera of Polymorphidae (Acanthocephala), inferred from nuclear and mitochondrial gene sequences. Mol Phylogenet Evol. 2013;68:176–84. 10.1016/j.ympev.2013.03.029 23567022

[22] García-Varela M, Nadler SA. Phylogenetic relationships of Palaeacanthocephala (Acanthocephala) inferred from SSU and LSU rDNA gene sequences. J Parasitol. 2005;91:1401–9. 16539024 10.1645/GE-523R.1

[23] García-Varela M, Hernández-Orts JS, López-Jiménez A, González-García MT. Molecular and morphological characterization of *Andracantha gravida* (Alegret, 1941) (Acanthocephala: Polymorphidae) in piscivorous birds from the Gulf of Mexico. J Helminthol. 2023;97:e31. 36960830 10.1017/S0022149X22000955

[24] Hernández-Orts JS, Lisitsyna OI, Kuzmina TA. First morphological and molecular characterization of cystacanths of *Corynosoma evae* Zdzitowiecki, 1984 (Acanthocephala: Polymorphidae) from Antarctic teleost fishes. Parasitol Int. 2022;91:102616. 35753653 10.1016/j.parint.2022.102616

[25] Lisitsyna O, Barčák D, Orosová M, Fan CK, Oros M. Acanthocephalans of marine and freshwater fishes from Taiwan with description of a new species. Folia Parasitol (Praha). 2023;70. 10.14411/fp.2023.021 38167244

[26] Presswell B, García-Varela M, Smales LR. Morphological and molecular characterization of two new species of Andracantha (Acanthocephala: Polymorphidae) from New Zealand shags (Phalacrocoracidae) and penguins (Spheniscidae) with a key to the species. J Helminthol. 2018;92:740–51. 29144212 10.1017/S0022149X17001067

[27] Ru SS, Yang RJ, Chen HX, Kuzmina TA, Spraker TR, Li L. Morphology, molecular characterization and phylogeny of *Bolbosoma nipponicum* Yamaguti, 1939 (Acanthocephala: Polymorphidae), a potential zoonotic parasite of human acanthocephaliasis. Int J Parasitol Parasites Wildl. 2022;18:212–20. 35783070 10.1016/j.ijppaw.2022.06.003 PMC9240962

[28] Santoro M, Palomba M, Gili C, Marcer F, Marchiori E, Mattiucci S. Molecular and morphological characterization of *Bolbosoma balaenae* (Acanthocephala: Polymorphidae), a neglected intestinal parasite of the fin whale *Balaenoptera physalus*. Parasitology. 2021;148:1293–302. 34100350 10.1017/S0031182021000925 PMC11010206

[29] Gazi M, Kim J, García-Varela M, Park C, Littlewood DTJ, Park JK. Mitogenomic phylogeny of Acanthocephala reveals novel class relationships. Zool Scr. 2016;45:437–54.

[30] Li DX, Yang RJ, Chen HX, Kuzmina TA, Spraker TR, Li L. Characterization of the complete mitochondrial genomes of the zoonotic parasites *Bolbosoma nipponicum* and *Corynosoma villosum* (Acanthocephala: Polymorphida) and the molecular phylogeny of the order Polymorphida. Parasitology. 2024;151:45–57. 37955106 10.1017/S0031182023001099 PMC10941042

[31] Pan TS, Nie P. The complete mitochondrial genome of *Pallisentis celatus* (Acanthocephala) with phylogenetic analysis of acanthocephalans and rotifers. Folia Parasitol (Praha). 2013;60:181. 23951925 10.14411/fp.2013.021

[32] Weber M, Wey-Fabrizius AR, Podsiadlowski L, Witek A, Schill RO, Sugár L, et al. Phylogenetic analyses of endoparasitic Acanthocephala based on mitochondrial genomes suggest secondary loss of sensory organs. Mol Phylogenet Evol. 2013;66:182–9. 23044398 10.1016/j.ympev.2012.09.017

[33] Delyamure SL. Helminthofauna of marine mammals (Ecology and Phylogeny). Akademiya Nauk SSSR. Moscow, Russia. Translated from Russian by Israel Program for Scientific Translations, Jerusalem, 1968; 1955.

[34] Meyer A. Acanthocephala. Pages 1–582 in H. G. Bronn, ed. Klassen und Ordnungen des Tier- Reichs. Akademishche Verlagsgesellschaft, Leipzig [In German]; 1932. 1–582 p.

[35] Petrochenko VI. Acanthocephala of domestic and wild animals. Vol. 1. Akademiya Nauk SSSR. Moscow, Russia. Translated from Russian by Israel Program for Scientific Translations, Jerusalem, 1971; 1958.

[36] Yamaguti S. Systema Helminthum. Vol. V. Acanthocephala. New York & London: Interscience Publishers, Inc.; 1963.

[37] WoRMS Editorial Board. World Register of Marine Species. Available from https://www.marinespecies.org at VLIZ. Accessed 2025–01–04 [Internet].

[38] Amin OM, Margolis L. Redescription of *Bolbosoma capitatum* (Acanthocephala: Polymorphidae) from false killer whale off Vancouver Island, with taxonomic reconsideration of the species and synonymy of *B. physeteris*. J-Helminthol Soc Wash. 1998;65:179–88.

[39] Abràmoff MD, Magalhães PJ, Ram SJ. Image processing with ImageJ. Biophotonics Int. 2004;11:36–42.

[40] Zur HI, von Japan A. Mem Fac Sci Agric Taihoku Imp Univ Formosa Jpn. 1935;14:7–23.

[41] Bankevich A, Nurk S, Antipov D, Gurevich AA, Dvorkin M, Kulikov AS, et al. SPAdes: a new genome assembly algorithm and its applications to single-cell sequencing. J Comput Biol. 2012;19:455–77. 22506599 10.1089/cmb.2012.0021 PMC3342519

[42] Bernt M, Donath A, Jühling F, Externbrink F, Florentz C, Fritzsch G, et al. MITOS: Improved *de novo* metazoan mitochondrial genome annotation. Mol Phylogenet Evol. 2013;69:313–9. 22982435 10.1016/j.ympev.2012.08.023

[43] Laslett D, Canbäck B. ARWEN: a program to detect tRNA genes in metazoan mitochondrial nucleotide sequences. Bioinformatics. 2008;24:172–5. 18033792 10.1093/bioinformatics/btm573

[44] Lagesen K, Hallin P, Rødland EA, Stærfeldt HH, Rognes T, Ussery DW. RNAmmer: consistent and rapid annotation of ribosomal RNA genes. Nucleic Acids Res. 2007;35:3100–8. 17452365 10.1093/nar/gkm160 PMC1888812

[45] Rozas J, Ferrer-Mata A, Sánchez-DelBarrio JC, Guirao-Rico S, Librado P, Ramos-Onsins SE, et al. DnaSP 6: DNA sequence polymorphism analysis of large data sets. Mol Biol Evol. 2017;34:3299–302. 29029172 10.1093/molbev/msx248

[46] Folmer O, Black M, Hoeh W, Lutz R, Vrijenhoek R. DNA primers for amplification of mitochondrial cytochrome c oxidase subunit I from diverse metazoan invertebrates. Mol Mar Biol Biotech. 1994;3:294–9. 7881515

[47] Sasaki M, Katahira H, Kobayashi M, Kuramochi T, Matsubara H, Nakao M. Infection status of commercial fish with cystacanth larvae of the genus *Corynosoma* (Acanthocephala: Polymorphidae) in Hokkaido. Japan Int J Food Microbiol. 2019;305:108256. 31299548 10.1016/j.ijfoodmicro.2019.108256

[48] Littlewood DTJ, Olson PD. Small subunit rDNA and the Platyhelminthes: signal, noise, conflict and compromise. In: Littlewood DTJ, Bray RA, editors. Interrelationships of the Platyhelminthes. Taylor & Francis; 2014. p. 262–78.

[49] Altschul SF, Gish W, Miller W, Myers EW, Lipman DJ. Basic local alignment search tool. J Mol Biol. 1990;215:403–10. 2231712 10.1016/S0022-2836(05)80360-2

[50] Tamura K, Nei M, Kumar S. Prospects for inferring very large phylogenies by using the neighbor-joining method. Proc Natl Acad Sci. 2004;101:11030–5. 15258291 10.1073/pnas.0404206101 PMC491989

[51] Tamura K, Stecher G, Kumar S. MEGA11: molecular evolutionary genetics analysis version 11. Mol Biol Evol. 2021;38:3022–7. 33892491 10.1093/molbev/msab120 PMC8233496

[52] Katoh K, Misawa K, Kuma K, Miyata T. MAFFT: a novel method for rapid multiple sequence alignment based on fast Fourier transform. Nucleic Acids Res. 2002;30:3059–66. 12136088 10.1093/nar/gkf436 PMC135756

[53] Castresana J. Selection of conserved blocks from multiple alignments for their use in phylogenetic analysis. Mol Biol Evol. 2000;17:540–52. 10742046 10.1093/oxfordjournals.molbev.a026334

[54] Nylander JAA. MrModeltest v2. Program distributed by the author. Vol. 2, Evolutionary Biology Centre Uppsala University. 2004. p. 1–2.

[55] Ronquist F, Teslenko M, van der Mark P, Ayres DL, Darling A, Höhna S, et al. MrBayes 3.2: efficient Bayesian phylogenetic inference and model choice across a large model space. Syst Biol. 2012;61(3):539–42. 10.1093/sysbio/sys029 PMC3329765 22357727

[56] Zwickl DJ. Genetic algorithm approaches for the phylogenetic analysis of large biological sequence datasets under the maximum likelihood criterion [PhD Thesis]. University of Texas; 2006.

[57] Kovalenko LM. Acanthocephalans of some fish in the tropical zone of the Pacific Ocean. Zool Zhurnal. 1981;60:1140–4

[58] Kita Y, Waki T, Kajihara H. Cystacanths of *Bolbosoma* (Acanthocephala: Polymorphidae) from six species of marine fish around Japan, with molecular information. Species Divers. 2024;29:317–25.

[59] Gatesy J, McGowen MR. Higher level phylogeny of baleen whales. In: George JC, Thewissen JGM, editors. The Bowhead Whale. Academic Press; 2021. p. 3–10.

[60] Andrade ALV, Pinedo MC, Barreto AS. Gastrointestinal parasites and prey items from a mass stranding of false killer whales, *Pseudorca crassidens*, in Rio Grande do Sul. Southern Brazil Rev Bras Biol. 2001;61:55–61. 10.1590/s0034-71082001000100008 11340462

[61] Odell DK, Asper ED, Cornell LH. A recurrent mass stranding of the false killer whale, *Pseudorca crassidens*. Florida Fish Bull. 1980;78:171–7.

[62] Pinto RM, Muniz-Pereira LC, Alves VC, Siciliano S. First report of a helminth infection for Bryde’s whale *Balaenoptera edeni* Anderson, 1878 (Cetacea, Balaenopteridae). Lat Am J Aquat Mamm. 2004;3:167–70.

[63] Williams EH, Bunkley-Williams L. Parasites of offshore big game fishes of Puerto Rico and the western Atlantic. [University of Puerto Rico]; 1996.

[64] Ahuir-Baraja AE. Parasites of the ocean sunfishes. In: The ocean sunfishes. CRC Press; 2020. p. 160–85.

[65] Hernandez-Orts JS, Timi JT, Raga JA, García-Varela M, Crespo EA, Aznar FJ. Patterns of trunk spine growth in two congeneric species of acanthocephalan: investment in attachment may differ between sexes and species. Parasitology. 2012;139:945–55. 22309658 10.1017/S0031182012000078

[66] Aznar FJ, Pérez-Ponce de León G, Raga JA. Status of *Corynosoma* (Acanthocephala: Polymorphidae) based on anatomical, ecological, and phylogenetic evidence, with the erection of *Pseudocorynosoman*. gen. J Parasitol. 2006;92:548–64. 16883999 10.1645/GE-715R.1

[67] Schmidt GD. *Andracantha*, a new genus of Acanthocephala (Polymorphidae) from fish-eating birds, with descriptions of three species. J Parasitol. 1975;61:615–20. 1165545

[68] Laskowski Z, Jeżewski W, Zdzitowiecki K. New data on the occurrence of Acanthocephala in Antarctic Amphipoda. Acta Parasitol. 2010;55:161–6.

[69] Zdzitowiecki K, Presler P. Occurrence of Acanthocephala in intermediate hosts, Amphipoda, in Admiralty Bay, South Shetland Islands. Antarctica Pol Polar Res. 2001;22:205–12.

[70] García-Varela M, de León GPP, Aznar FJ, Nadler SA. Systematic position of *Pseudocorynosoma* and *Andracantha* (Acanthocephala, Polymorphidae) based on nuclear and mitochondrial gene sequences. J Parasitol. 2009;95:178–85. 18576894 10.1645/GE-1538.1

[71] Van Cleave HJ. A preliminary analysis of the acanthocephalan genus *Corynosoma* in mammals of North America. J Parasitol. 1953;39:1–13. 13035576

