# Supplementary material for: Revisiting the genus Bolbosoma Porta, 1908 (Acanthocephala: Polymorphidae): host specificity, phylogeny, and species synonymization
Source: Parasit Vectors. 2025 Sep 24;18:392. doi: 10.1186/s13071-025-07015-3 (PMC12462197; doi:10.1186/s13071-025-07015-3)
Supplement: Supplementary file 1 — Table S1. List of Bolbosoma species and potential definitive hosts, indicating maturity stage of specimens. [file 13071_2025_7015_MOESM1_ESM.docx]

**Table S1**. List of Bolbosoma species and potential definitive hosts, indicating maturity stage of specimens. The presence of adults and corresponding references are highlighted in bold.

| ***Bolbosoma* species**  **(accepted name)** | **Host species** | **Maturity stage of *Bolbosoma* specimens** | **References** |
| --- | --- | --- | --- |
| *Bolbosoma australis* | *Balaenoptera borealis* | **Adult** | **89, 105** |
|  | *Eubalaena australis* | Immature | 89, 105 |
| *Bolbosoma balaenae* | *Balaena mysticetus* | Not specified | 14, 26, 65, 72, 100 |
|  | *Balaenoptera acutorostrata* | Not specified | 12, 14, 26, 35, 65, 72, 77, 90, 100 |
|  | *Balaenoptera borealis* | Not specified | 14, 26, 65, 72, 77, 100, 105 |
|  | *Balaenoptera musculus* | Not specified | 14, 26, 64, 72, 100 |
|  | *Balaenoptera physalus* | **Adult** | 26, 35, **83**, 100, 105 |
|  | *Eschrichtius robustus* | **Adult** | **25** |
|  | *Eubalaena australis* | Not specified | 100 |
|  | *Megaptera novaeangliae* | **Adult** | 14, **17**, 26, **45**, 65, 72, 77, **80**, 90, 100 |
|  | *Globicephala melas* | Not specified | 90 |
|  | *Hyperoodon ampullatus* | Not specified | 26, 65, 72, 77, 100 |
|  | *Stenella attenuata* | Not specified | 22 |
|  | *Stenella coeruleoalba* | Immature | 34 |
|  | *Stenella longirostris* | Not specified | 22 |
|  | *Eudyptula novaehollandiae* | Immature | 15, 16 |
| *Bolbosoma brevicolle* | *Balaenoptera acutorostrata* | **Adult** | 14, 26, 29, 65, 72, **76**, 77, 100 |
|  | *Balaenoptera borealis* | Immature or not specified | 14, 26, 29, 65, 72, 100, 105 |
|  | *Balaenoptera musculus* | **Adult** | 13, 14, 26, 29, 35, 64, 65, 72, 77, **87**, 100, **105** |
|  | *Balaenoptera physalus* | Not specified | 13, 14, 26, 29, 35, 65, 72, 100 |
|  | *Eubalaena japonica* | Not specified | 26 |
|  | *Physeter macrocephalus* | Not specified | 14, 26, 29, 100, 102 |
| *Bolbosoma caenoforme* | *Uria aalge* | Not specified | 46, 102 |
| *Bolbosoma capitatum* | *Balaenoptera edeni* | **Adult** | **73** |
|  | *Balaenoptera musculus* | Not specified | 90 |
|  | *Balaenoptera physalus* | Not specified | 56 |
|  | *Globicephala macrorhynchus* | **Adult** | **5**, 18, 66, **91**, 99 |
|  | *Globicephala melas* | **Adult** | **5**, **9**, **10**, **11**, 14, 21, 26, 29, **30**, 34, 35, 43, **54**, 59, 62, 65, **70**, 72, 77, **79**, 90, 100, 102 |
|  | *Lagenorhynchus albirostris* | Not specified | 84 |
|  | *Orcinus orca* | Not specified | 26, 29, 36, 37, 72, 100, 102 |
|  | *Peponocephala electra* | **Adult** | 18, **91** |
|  | *Phocoena phocoena* | Not specified | 37 |
|  | *Physeter macrocephalus* | **Adult** | **5**, 14, 24, **26**, 29, **38**, **42**, 44, 72, 90, 100, 102 |
|  | *Pseudorca crassidens* | **Adult** | **5**, 7, 14, 19, 26, 29, **31**, 35, **47**, **48**, 65, 69, 72, 76, 77, 90, 100, 102 |
|  | *Stenella coeruleoalba* | Immature | 34, 81 |
|  | *Steno bredanensis* | Immature or not specified | 5, 13, 14, 26, 29, 35, 65, 72, 100, 102 |
|  | *Arctocephalus forsteri* | Not specified | 90 |
|  | *Arctocephalus pusillus* | Not specified | 90 |
|  | *Homo sapiens* | Immature | 8 |
| *Bolbosoma hamiltoni* | *Balaenoptera musculus* | Not specified | 13, 14, 26, 35, 64, 72, 100, 102, 105 |
|  | *Balaenoptera physalus* | **Adult** | 13, 14, 17, 26, 35, 72, 100, 102, **105** |
|  | *Stenella longirostris* | Immature | 2 |
| *Bolbosoma nipponicum* | *Balaenoptera acutorostrata* | **Adult** | 26, 72, 100, **101**, **102** |
|  | *Balaenoptera borealis* | Not specified | 26, 72, 100, 102 |
|  | *Balaenoptera physalus* | Not specified | 26, 72, 100, 102 |
|  | *Balaenoptera musculus* | Not specified | 64, 100 |
|  | *Eubalaena japonica* | Not specified | 100 |
|  | *Megaptera novaeangliae* | Not specified | 100 |
|  | *Orcinus orca* | Not specified | 100 |
|  | *Callorhinus ursinus* | Immature or not specified | 23, 26, 28, 50, 52, 53, 72, 82, 102 |
|  | *Erignathus barbatus* | Immature | 93 |
|  | *Eumetopias jubatus* | Immature or not specified | 23, 26, 50, 72, 85, 102, 103 |
|  | *Histriophoca fasciata* | Immature or not specified | 74 |
|  | *Phoca largha* | Not specified | 51 |
|  | *Phoca vitulina* | Not specified | 49 |
|  | *Pusa hispida* | Immature or not specified | 1, 23, 26, 50, 72, 75, 102 |
|  | *Larus crassirostris* | Not specified | 50 |
| *Bolbosoma scomberomori* | *Delphinus delphis* | Immature | 97 |
| *Bolbosoma tuberculata* | *Balaenoptera borealis* | **Adult** | **33**, **88**, 100, **106** |
|  | *Balaenoptera edeni* | **Adult** | **88**, 100, **106** |
|  | *Balaenoptera physalus* | Immature | 106 |
|  | *Physeter macrocephalus* | Immature | 88, 106 |
| *Bolbosoma turbinella* | *Balaenoptera borealis* | **Adult** | 13, 14, 24, 29, 26, **33**, 35, **40**, **57**, 58, 65, 67, **68**, 72, **76**, 77, **89**, 100, 102, **104** |
|  | *Balaenoptera edeni* | **Adult** | **68** |
|  | *Balaenoptera musculus* | **Adult** | 4, 14, 26, 29, **63**, 64, 65, 72, 77, 89, 90, 98, 100, 102 |
|  | *Balaenoptera physalus* | Immature or not specified | 26, 29, 72, 89, 100, 102 |
|  | *Eubalaena australis* | Not specified | 100 |
|  | *Eubalaena japonica* | Immature or not specified | 26, 29, 72, 89, 100, 102 |
|  | *Megaptera novaeangliae* | Immature or not specified | 14, 26, 29, 35, 65, 72, 89, 100, 102 |
|  | *Hyperoodon ampullatus* | Immature or not specified | 14, 26, 29, 65, 72, 77, 89, 98, 100, 102 |
|  | *Physeter macrocephalus* | Not specified | 29, 100 |
|  | *Pontoporia blainvillei* | **Adult** | 6, **86** |
|  | *Stenella coeruleoalba* | Immature | 81 |
|  | *Arctocephalus australis* | Immature | 96 |
| *Bolbosoma vasculosum* | *Delphinus delphis* | Immature or not specified | 4, 14, 20, 26, 27, 65, 72, 77, 91, 95, 100, 102 |
|  | *Feresa attenuata* | Not specified | 66 |
|  | *Kogia breviceps* | Immature or not specified | 61, 71 |
|  | *Mesoplodon bidens* | Immature or not specified | 4, 14, 26, 72, 95, 100, 102 |
|  | *Mesoplodon bowdoini* | Not specified | 90 |
|  | *Mesoplodon densirostris* | Not specified | 18 |
|  | *Stenella attenuata* | Not specified | 22 |
|  | *Stenella coeruleoalba* | Immature or not specified | 3, 34, 55, 60, 78, 91 |
|  | *Stenella frontalis* | Not specified | 66 |
|  | *Stenella longirostris* | Not specified | 22 |
|  | *Tursiops truncatus* | **Adult** | **92** |
|  | *Ziphius cavirostris* | Immature | 32 |
|  | *Arctocephalus pusillus* | **Adult** | **39**, 90 |
|  | *Scomberomorus cavalla* | **Adult** | **99** |
|  | *Thunnus thynnus* | **Adult** | **41** |
|  | *Caretta caretta* | Immature | 94 |

**References**

1. Adams, A. M. (1988). Taxonomy, systematics and ecology of helminth parasites of the ringed seal, *Phoca hispida* Schreber, in Alaskan waters. PhD Thesis (University of Washington).
2. Aguilar-Aguilar, R., Moreno-Navarrete, Salgado-Maldonado, G., and Villa-Ramírez, B. (2001). Gastrointestinal helminths of spinner dolphins *Stenella longirostris* (Gray, 1828) (Cetacea: Delphinidae) stranded in La Paz Bay, Baja California Sur, Mexico. *Comparative Parasitology, 68*(2), 272–274.
3. Agustí, C., Aznar, F. J., and Raga, J. A. (1999). Intestinal helminth communities of Mediterranean striped dolphin. Paper presented at the 13th Biennial Conference on the Biology of Marine Mammals. Hawaii, November 28 - December 3.
4. Amin, O. M. (1998) Marine flora and fauna of the eastern United States – Acanthocephala. *NOAA Technical Report NMFS*, *135*, 1-27.
5. Amin, O. M., and Margolis, L. (1998). Redescription of *Bolbosoma capitatum* (Acanthocephala: Polymorphidae) from false killer whale off Vancouver Island, with taxonomic reconsideration of the species and synonymy of *B. physeteris*. *Journal of the Helminthological Society of Washington*, *65*(2), 179–188.
6. Andrade, A., Pinedo, C., and Pereira, J. (1997). The gastrointestinal helminths of the Franciscana, *Pontoporia blainvillei*, in Southern Brazil. *Reports of the International Whaling Commission, 47*, 669–673.
7. Andrade, A., Pinedo, C., and Barreto, A. S. (2001). Gastrointestinal parasites and prey items from a mass stranding of false killer whales, *Pseudorca crassidens*, in Rio Grande do Sul, Southern Brazil. *Revista Brasileira de Biologia*, *61*, 55–61.
8. Arizono, N., Kuramochi, T., and Kagei, N. (2012). Molecular and histological identification of the acanthocephalan *Bolbosoma* cf. *capitatum* from the human small intestine. *Parasitology International*, *61*(4), 715-718.
9. Balbuena, J. A. (1991). Estudio taxonómico y ecológico de la parasitofauna del Calderón común, *Globicephala melas* (Traill, 1809), en las aguas de Europa. PhD Thesis (Universitat de València) [in Spanish].
10. Balbuena, J. A., and Raga, J. A. (1993). Intestinal helminth communities of the long-finned pilot whale (*Globicephala melas*) off the Faroe Islands. *Parasitology*, *106*(3), 327-333.
11. Balbuena, J. A., and Raga, J. A. (1994). Intestinal helminths as indicators of segregation and social structure of pods of long-finned pilot whales (*Globicephala melas*) off the Faroe Islands. *Canadian Journal of Zoology*, *72*(3), 443–448.
12. Baylis, H. A. (1928) Records of some parasitic worms from British Vertebrates. *Annals and Magazine of Natural History,* *10*(1), 329-343.
13. Baylis, H. A. (1929). Parasitic Nematoda and Acanthocephala collected in 1925-1927. *Discovery Reports*, *1*, 541–559.
14. Baylis, H. A. (1932). A list of worms parasitic in Cetacea. *Discovery Reports,* *6*, 393–418
15. Bennett, J., McPherson, O., and Presswell, B. (2021). Gastrointestinal helminths of little blue penguins, *Eudyptula novaehollandiae* (Stephens), from Otago, New Zealand. *Parasitology international*, *80*, 104185.
16. Bennett, J., Poulin, R., and Presswell, B. (2023). Acanthocephalan diversity and host associations revealed from a large-scale biodiversity survey. *Diversity*, *15*(5), 665.
17. Cockrill, W. R. (1960) Pathology of the Cetacea. A veterinary study on whales. Part I. *British Veterinary Journal*, *116*(4), 133-144.
18. Colón-Llavina, M. M., Mignucci-Giannoni, A., Mattiucci, S., Paoletti, M., Nascetti, G., and Williams, E. H. (2009). Additional records of metazoan parasites from Caribbean marine mammals, including genetically identified anisakid nematodes. *Parasitology Research*, *105*, 1239–1252.
19. Colón-Llavina, M. M., Mattiucci, S., Nascetti, G., Harvey, J. T., Williams, E. H., and Mignucci-Giannoni, A. A. (2019). Some metazoan parasites from marine mammals stranded in California. *Pacific Science*, *73*(4), 461-473.
20. Costa, G., Chubb, J. C., and Veltkamp, C. J. (2000). Cystacanths of *Bolbosoma vasculosum* in the black scabbard fish *Aphanopus carbo*, oceanic horse mackerel *Trachurus picturatus* and common dolphin *Delphinus delphis* from Madeira, Portugal. *Journal of Helminthology*, *74*(2), 113-120.
21. Cowan, Daniel F. (1967) Helminth parasites of the pilot whale *Globicephala melaena* (Traill 1809). *Journal of Parasitology*, *53*(1), 166-167.
22. Dailey, M. D., and Perrin, W. F. (1973). Helminth parasites of porpoises of the genus *Stenella* in the Eastern Tropical Pacific, with descriptions of two new species: *Mastigonema stenellae* gen.et.es.n. (Nematoda: Spiruroidea) and *Zalophotrema pacificum* sp. n. (Trematoda: Digenea). *Fishery Bulletin*, 71(2), 455-471.
23. Dailey, M. D. (1975). The distribution and intraspecific variation of helminth parasites in pinnipeds. *Rapports et procès-verbaux des réunions*, *169*, 338-352.
24. Dailey, M. D., and Vogelbein, W. K. (1991). Parasite fauna of three species of Antarctic whales with reference to their use as potential stock indicators. *Fishery Bulletin*, *89*(3), 355–365.
25. Dailey, M. D., Gulland, F. M. D., Lowenstine, L. J., Silvagni, P., and Howard, D. (2000). Prey, parasites and pathology associated with the mortality of a juvenile gray whale (Eschrichtius robustus) stranded along the northern California coast. *Diseases of Aquatic Organisms*, *42*(2), 111-117.
26. Delyamure, S. L. (1955). *Helminthofauna of marine mammals (Ecology and Phylogeny)*. Moscow, Russia: Akademiya Nauk SSSR.
27. Delyamure, S. L. (1957). Dependence of the helminth fauna of dolphins of the Black and Azov seas on ecologico-geographical factors. *Fisheries Research Board of Canada*, *131*, 1–10.
28. Delyamure, S. L., and Skryabin A. S. (1960). Helminthofauna of seals, *Callorhinus ursinus*, on Komandorskie Isles. *Scientific Reports of Higher Schools-Biological Sciences*, *2*, 11-14 [in Russian].
29. Delyamure, S. L., and Skrjabin, A. S. (1972). Helminths. In Berzin A. A. (Ed.), *The Sperm Whale* (pp. 278–304). Jerusalem: Israel Program for Scientific Translations.
30. Edmonds, S. J. (1956). Australian Acanthocephala No. 10. *Transactions of the Royal Society of South Australia*, *80*, 76-80.
31. Edmonds, S. J. (1987). A note on the occurrence of *Bolbosoma capitatum* (Linstow, 1880) (Acanthocephala) from a false killer whale stranded on the coast of Western Australia. *Records of the Western Austalian Museum*, *13*, 317–318.
32. Fernández, M., Aznar, F. J., Montero, F. E., Georgiev, B. B., and Raga, J. A. (2004). Gastrointestinal helminths of Cuvier's beaked whales, *Ziphius cavirostris*, from the western Mediterranean. *Journal of Parasitology*, *90*(2), 418-420.
33. Galkina, G. G. (1972). On the monorchic Acanthocephala from the genus *Bolbosoma* Porta, 1908 (Acanthocephala: Polymorphidae), *Trudy Biologii Pochvennogo Instituta, Vladivostok,* *11*(114), 116-120 [in Russian].
34. García-Gallego, A., Raga, J. A., Fraija-Fernández, N., and Aznar, F. J. (2023). Temporal and geographical changes in the intestinal helminth fauna of striped dolphins, *Stenella coeruleoalba*, in the western Mediterranean: a long-term analysis (1982-2016). *Frontiers in Marine Science*, *10*, 1272353.
35. Gibson, D. I., and Harris, E. A. 1979. The helminth-parasites of cetaceans in the collection of the British Museum (Natural History). *Investigations on Cetacea*, *10*, 309–324.
36. Gibson, D. I., and Bray, R. A. (1997). *Oschmarinella albamarina* (Treschev, 1968) n. comb., a liver fluke from the killer whale *Orcinus orca* (L.) off the British coast. *Systematic Parasitology*, *36*(1), 39-46.
37. Gibson, D. I., Harris, E. A., Bray, R. A., Jepson, P. D., Kuiken, T., Baker, J. R., and Simpson, V. R. (1998). A survey of the helminth parasites of cetaceans stranded on the coast of England and Wales during the period 1990-1994. *Journal of Zoology*, *244*(4), 563-574.
38. Gubanov, N. M. (1952). Helminthic fauna of commercial animals of the Sea of Okhotsk and Pacific Ocean. *Vsesoyuznyi Institut Gel'mintologii imeni K. I. Skryabina*, *7*, 380-381 [in Russian].
39. Halajian, A., Smales, L., Heckmann, R., Amakali, A. M., Tjipute, M., Wilhelm, M. R., and Luus-Powell, W. J. (2020). *Corynosoma australe* and *Bolbosoma vasculosum* (Polymorphidae: Acanthocephala) from *Arctocephalus pusillus pusillus* (Otariidae) and *Argyrosomus* spp. (Sciaenidae) from the Namibian Coast of Africa. *Comparative Parasitology*, *87*(1), 127-134.
40. Harada, I. (1931). Das Nervensystem von *Bolbosoma turbinella* (Dies.). *Japanese Journal of Zoology*, *3*, 161-199 [in German].
41. Harada, I. (1935). Zur Acanthocephalenfauna von Japan. *Memoirs of the Faculty of Science and Agriculture, Taihoku Imperial University*, *14*(2), 7-23 [in German].
42. Hoberg, E. P., Daoust, P. Y., and McBurney, S. (1993). *Bolbosoma capitatum* and *Bolbosoma* sp.(Acanthocephala) from sperm whales (*Physeter macrocephalus*) stranded on Prince Edward Island, Canada. *Journal of the Helminthological Society of Washington*, *60*(2), 205-210.
43. IJsseldijk, L. L., Leopold, M. F., Bravo-Rebolledo, E. L., Deaville, R., Haelters, J., IJzer, J., Jepson, P. D., and Gröne, A. (2015). Fatal asphyxiation in two long-finned pilot whales (*Globicephala melas*) caused by common soles (*Solea solea*). *PloS one*, *10*(11), e0141951.
44. IJsseldijk, L. L., Van Neer, A., Deaville, R., Begeman, L., van de Bildt, M., van den Brand, J. M. A., Brownlow, A., ... and Siebert, U. (2018). Beached bachelors: An extensive study on the largest recorded sperm whale *Physeter macrocephalus* mortality event in the North Sea. *PLoS One*, *13*(8), e0201221.
45. Johnston, T. H., and Effie W. D. (1929). Australian Acanthocephala, No. 1. Census of recorded hosts and parasites. *Transactions and Proceedings of the Royal Society of South Australia*, *53*, 146-154.
46. Kihara, M. (1959). Studies on the Acanthocephala (V). An Acanthocephala of *Uria aalge inornata* Salomonsen of northern ocean. *Proceedings of Japanese Parasitological Society (East Japan Section)*, *19*, 30 [in Japanese].
47. Kikuchi, S., and Nakajima M. (1991). Morphology and pathogenicity of Acanthocephala parasitic in the small intestine of false killer whale. *Journal of Bengal Natural History Society*, *40*, 123 [in Japanese].
48. Kikuchi, S., and Nakajima M. (1993). *Bolbosoma capitatum* (Acanthocephala) from false killer whales, *Pseudorca crassidens*, and its pathogenicity. *Japanese Journal of Parasitology*, *42*(5), 398-408, [in Japanese].
49. Kovalenko, L. M. (1975). The helminth fauna of Otariidae seals from the Kuril Islands. In: Agarkov, G. B., Arsenev, V. A., Zemskii, V. A., Smelova, I. V., Sokolov, V. E., Sokolov, A. S., Tveryanovich, V. A., Tomilin, A. G., and Yablokov, A. V. (Eds.), *Morskie Mlekopitayuschchie, Materialy Vsesoyuznogo Soveshchaniya po Mlekopitayushchim, Chast' 2 (Materialy VI Vesoyuznogo Obshchestva, Kiev, Oktybr' 1975)* (pp. 137-139). Kiev, USSR, Naukova Dumka [in Russian].
50. Krotov, A. I., and Delyamure S. L. (1952). On the helminth fauna of mammals and birds of the USSR. *Trudy Gel'minty Laboratorii, Akademiya Nauk SSSR*, *6*, 278-292 [in Russian]
51. Krotov, A. I. (1960). Zoogeographical analysis and hypothesis on the origin of helminth fauna of vertebrates on Sakhalin Island. *Zoologicheskiï Zhurnal*, *39*(4), 481-489 [in Russian].
52. Kuzmina, T. A., Lisitsyna, O. I., Lyons, E. T., Spraker, T. R., and Tolliver, S. C. (2012). Acanthocephalans in northern fur seals (*Callorhinus ursinus*) and a harbor seal (*Phoca vitulina*) on St. Paul Island, Alaska: species, prevalence, and biodiversity in four fur seal subpopulations. *Parasitology Research*, *111*, 1049-1058.
53. Kuzmina, T. A., Kuzmin, Y., Dzeverin, I., Lisitsyna, O. I., Spraker, T. R., Korol, E. M., and Kuchta, R. (2021). Review of metazoan parasites of the northern fur seal (*Callorhinus ursinus*) and the analysis of the gastrointestinal helminth community of the population on St. Paul Island, Alaska. *Parasitology Research*, *120*, 117-132.
54. Machado Filho, D. A. (1964) Contribution to the knowledge of the genus Bolbosoma Porta, 1908 (Palaeacanthocephala, Polymorphidae). *Revista Brasileira de Biologia*, 24(3), 341-348 [in Portuguese]
55. Manfredi, M. T., Dini, W., Ganduglia, S., Podesta, M., and Repetto, G. (1992). Parasitological findings in striped dolphins *Stenella coeruleoalba*. *European Research on Cetaceans*, *6*, 218–219.
56. Marcer, F., Marchiori, E., Centelleghe, C., Ajzenberg, D., Gustinelli, A., Meroni, V., and Mazzariol, S. (2019). Parasitological and pathological findings in fin whales *Balaenoptera physalus* stranded along Italian coastlines. *Diseases of Aquatic Organisms*, *133*(1), 25-37.
57. Margolis, L., and Pike, G. C. (1955). Some helminth parasites of Canadian pacific whales. *Journal of the Fisheries Research Board of Canada*, *12*, 97–120.
58. Margolis, L., and Dailey, M. D. (1972). Revised annotated list of parasites from sea mammals caught off the west coast of North America. *NOAA Technical Report*, *647*, 1–23.
59. Margolis, L., and Arai, H. P. (1989). Synopsis of the parasites of vertebrates of Canada: parasites of marine mammals. *Alberta Agriculture*, *Animal Health Division*, 26 pp.
60. Mateu, P., Raga, J. A., Fernandez, M., and Aznar, F. J. (2014). Intestinal helminth fauna of striped dolphins (*Stenella coeruleoalba*) in the western Mediterranean: No effects of host body length, age and sex. *Marine Mammal Science*, *30*(3), 961–977.
61. McAlpine, D. F., Murison, L. D., and Hoberg, E. P. (1997). New records for the pygmy sperm whale, *Kogia breviceps* (Physeteridae) from Atlantic Canada with notes on diet and parasites. *Marine Mammal Science*, *13*(4), 701-704.
62. McManus, T. J., Wapstra, J. E., Guiler, E. R., Munday, B. L., and Obendorf, D. L. (1984). Cetacean strandings in Tasmania from February 1978 to May 1983. In *Papers and Proceedings of the Royal Society of Tasmania* (Vol. 118, pp. 117-135).
63. Measures, L. N. (1992). *Bolbosoma turbinella* (Acanthocephala) in a blue whale, *Balaenoptera musculus*, stranded in the St. Lawrence estuary, Quebec. *Journal of the Helminthological Society of Washington*, *59*(2), 206-211.
64. Measures, L. N. (1993). Annotated list of metazoan parasites reported from the blue whale, *Balaenoptera musculus*. *Journal of the Helminthological Society of Washington*, *60*(1), 62-66.
65. Meyer, A. (1932). Acanthocephala. In Bronns, H. G. (Ed.), *Klassen und Ordnungen des Tier-Reichs* (pp. 1-332). Leipzig [in German].
66. Mignucci-Giannoni, A., Hoberg, E. P., Siegel-Causey, D., and Williams, E. H. (1998). Metazoan parasites and other symbionts of cetaceans in the Caribbean. *Journal of Parasitology*, *84*(5), 939–946.
67. Muniz-Pereira, L. C., Vicente, J. J., and Noronha, D. (1999). Helminth parasites of whales in Brazil. *Revista Brasileira de Zoologia*, *16*, 249–252.
68. Muñoz, G., and Olmos, V. (2008). Revisión bibliográfica de especies endoparásitas y hospedadoras de sistemas acuáticos de Chile. *Revista de Biología Marina y Oceanografía*, *43*(2), 173–245 [in Spanish].
69. Odell, D. K., Asper, E. D., Baucom, J., and Cornell, L. H. (1980). A recurrent mass stranding of the false killer whale, *Pseudorca crassidens*, in Florida. *Fishery Bulletin*, *78*(1), 171-177.
70. Parona, C. (1893). Sopra una straordinaria polielmintiasi da echinorinco (*Echinorh. capiatus*) nel *Globicephalus svinerai* Flow., pescato nel mare di Genova. *Atti della Società Ligustica di Scienze Naturali e Geografiche*, *4*, 314-324 [in Italian].
71. Pendergraph, G. E. (1971). First report of the acanthocephalan, *Bolbosoma vasculosum* (Rudolphi, 1819), from the pygmy sperm whale, *Kogia breviceps*. *Journal of Parasitology*, *57*(5), 1109.
72. Petrochenko, V. I. (1958). *Acanthocephala of domestic and wild animals* (Vol. 1). Akademiya Nauk SSSR. Moscow, Russia. Translated from Russian by Israel Program for Scientific Translations, Jerusalem, 1971.
73. Pinto R. M., Muniz-Pereira, L. C., Alves, V. C., and Siciliano, S. (2004). First report of a helminth infection for Bryde's whale, *Balaenoptera edeni* Anderson, 1878 (Cetacea, Balaenopteridae). *Latin American Journal of Aquatic Mammals*, *3*, 167-170.
74. Popov, V. N. (1975). New data on the helminth fauna of *Histriophoca fasciata* Zimm. from the southern part of the Okhotsk Sea. *Parazitologiya*, *9*(1), 31-36 [in Russian].
75. Popov, V. N., Jurakhno, M. V., and Skryabin V. A. (1980). Helminth fauna of the Okhotsk ringed seal from the Patience Bay and Laperouse Strait. *Parazitologiya*, *14*(1), 48-55 [in Russian].
76. Porta, A. (1908). Gli acanthocefali dei mammiferi. *Archivo Zoologico, Napoli*, *4*(1), 239-285 [in Italian].
77. Porta, A. (1908). Gli acantocefali dei mammiferi. Noto preventiva. *Archives de Parasitologie, Paris*, *12*(2), 268-282 [in Italian].
78. Raga, J. A., and Carbonell, E. (1985). New data about parasites on *Stenella coeruleoalba* (Meyer, 1833) (Cetacea: Delphinidae) in the western Mediterranean Sea. *Investigations on Cetacea*, *17*, 207–213.
79. Raga, J. A., and Balbuena, J. A. (1993). Parasites of the long-finned pilot whale, *Globicephala melas* (Traill, 1809), in European waters. *Report of the International Whaling Commission*, *14*, 391–406.
80. Rees, G. (1953). A record of some parasitic worms from whales in the Ross Sea. *Parasitology*, *43*, 27–34.
81. Rosas, F. C. W., Monteiro-Filho, E. L. A., Marigo, J., Santos, R. A., Andrade, A. L. V., Rautenberg, M., Oliveira, M. R., and Bordignon, M. O. (2002). The striped dolphin, *Stenella coeruleoalba* (Cetacea: Delphinidae), on the coast of Sao Paulo State, southeastern Brazil. *Aquatic Mammals*, *28*, 60-66.
82. Ru, S. S., Yang, R. J., Chen, H. X., Kuzmina, T. A., Spraker, T. R., and Li, L. (2022). Morphology, molecular characterization and phylogeny of *Bolbosoma nipponicum* Yamaguti, 1939 (Acanthocephala: Polymorphidae), a potential zoonotic parasite of human acanthocephaliasis. *International Journal for Parasitology: Parasites and Wildlife*, *18*, 212-220.
83. Santoro, M., Palomba, M., Gili, C., Marcer, F., Marchiori, E., and Mattiucci, S. (2021). Molecular and morphological characterization of *Bolbosoma balaenae* (Acanthocephala: Polymorphidae), a neglected intestinal parasite of the fin whale *Balaenoptera physalus*. *Parasitology*, *148*(11), 1293-1302.
84. Schick, L., IJsseldijk, L. L., Grilo, M. L., Lakemeyer, J., Lehnert, K., Wohlsein, P., Ewers, C., ... and Siebert, U. (2020). Pathological findings in white-beaked dolphins (*Lagenorhynchus albirostris*) and Atlantic white-sided dolphins (*Lagenorhynchus acutus*) from the south-eastern North Sea. *Frontiers in veterinary science*, *7*, 262.
85. Shults, L. M. (1986). Helminth parasites of the Steller sea lion, *Eumetopias jubatus*, in Alaska. *Proceedings of the Helminthological Society of Washington*, *53*(2), 194-197.
86. Silva, R. Z. and Cousin, J. C. B. (2006). Anormalidade intestinal parasitaria em *Pontoporia blainvillei* (Cetacea, Platanistoidea, Pontoporiidae) da regiao litoranea de Rio Grande, RS, Brasil. *Biociências*, *14*(1), 37-46 [in Portuguese].
87. Skryabin, A. S. (1959). New species of helminths from marine mammals from the Pacific Ocean and Far-eastern Sea. *Izvestiya Crimskogo-Pokagogich Eskogo Instuta*, *34*, 99-118 [in Russian].
88. Skrjabin, A. S. (1970). A new species of acanthocephalans, *Bolbosoma tuberculata* sp. n. (Fam. Polymorphidae Meyer, 1931) - a parasite of whales. *Parazitologiya*, *4*, 334–337 [in Russian].
89. Skryabin, A. S. (1972). Morphological differences between *Bolbosoma turbinella* (Diesing, 1851) (fam. Polymorphidae) Acanthocephala from the Northern and Southern Hemispheres. *Parazitologiya*, 6(1), 57-64 [in Russian].
90. Smales, L. R. (2003). An annotated checklist of the Australian Acanthocephala from mammalian and bird hosts. *Records of the South Australian Museum*, *36*(1), 59-82.
91. Suárez-González, Z., González, J. F., Arbelo, M., Sierra, E., Castro-Alonso, A., Hernández, J. N., Martín, V., ... and Fernández, A. (2024). Parasitic Infections in Stranded Whales and Dolphins in Canary Islands (2018–2022): An Update. *Animals*, *14*(23), 3377.
92. Terracciano, G., Fichi, G., Comentale, A., Ricci, E., Mancusi, C., and Perrucci, S. (2020). Dolphins stranded along the tuscan coastline (Central Italy) of the “Pelagos sanctuary”: A parasitological investigation. *Pathogens*, *9*(8), 612.
93. Treshchev, V. V. and Popov, V. N. (1972). Helminths of *Erignathus barbatus nauticus* from Tauiskaya Bay, Okhotsk Sea (USSR). In: Markevich, A. P. (Ed.), *Problemy Parazitologii, Trudy Nauchnoi Konferentsii Parazitologov Ukrainskoi SSR, 7th, Part 1* (pp. 338-340). Kiev, USRR, Naukova Dumka [in Russian].
94. Valente, A. L., Delgado, C., Moreira, C., Ferreira, S., Dellinger, T., Pinheiro de Carvalho, M. A., and Costa, G. (2009). Helminth component community of the loggerhead sea turtle, *Caretta caretta*, from Madeira Archipelago, Portugal. *Journal of Parasitology*, *95*(1), 249-252.
95. Van Cleave, H. J. (1953). Acanthocephala of North American mammals. *Illinois Biological Monographs*, *23*, 1–179.
96. Vivian, I. F., Perin, P. P., Amorim, D. B. D., Benatti, D., Tebaldi, J. H., and Hoppe, E. G. L. (2023). Helminths of South American fur seals (*Arctocephalus australis*) from the subtropical convergence zone of the southwestern Atlantic. *Revista Brasileira de Parasitologia Veterinária*, *32*(1), e014522.
97. Wang, P. Q. (1980). Notes on the Acanthocephala from Fugian. II. *Acta Zootaxonomica Sinica*, *2*, 116-123 [in Chinese].
98. Wesenberg-Lund, E. (1926). Conspectus Faunae Groenlandicae. Acanthocephaler. *Meddelanden Om Gruenlandicae, Supplement*, *23*, 145-155 [in Danish].
99. Williams, E. H., and Bunkley-Williams L. (1996). Acanthocephala (Spiny-Headed Worms), In: *Parasites of Offshore Big Game Fishes of Puerto Rico and the Western Atlantic* (pp. 156-162). San Juan and Mayaguez, Puerto Rico, Department of Natural and Environmental Resources and University of Puerto Rico.
100. Yablokov, A. V., Bel’kovich, V. M., and Borisov, V. I. (1972). *Whales and dolphins, part I*. Moscow: Kity I Del’finy. Nauk SSSR.
101. Yamaguti, S. (1939). Studies on the helminth fauna of Japan. Part 29. Acanthocephala, II. *Japanese Journal of Zoology*, *8*(3), 317–351.
102. Yamaguti, S. (1963). *Systema Helminthum Vol. V Acanthocephala*. New York: Interscience.
103. Yurakhno, M.V., Loboda, A.P., Stryukov, A. A., Solov’ov, V. V. (2005). About validity of species *Bolbosoma bobrovi* (Acanthocephala, Polymorphidae). *Vestnik Zoology: Supplement*, *19*, 371–373 [in Russian].
104. Zakariah, M. I., Ahmad, M. T. A., Razak, M, S., Husin, N. M., Wahab, W., Abdullah, R. A. A., Yusoff, N. A. H., Agos, S., and Hassan, M. (2021). Morphological Identification of *Bolbosoma turbinella* (Acanthocephala) in *Balaenoptera borealis* (Sei Whale) from Straits of Malacca, Malaysia. *Jurnal Ilmiah Perikanan dan Kelautan*, *13*(1), 73–80.
105. Zdzitowiecki, K. (1986). Acanthocephala of the Antarctic, *Polish Polar Research*, *7*(1-2), 79-117.
106. Zdzitowiecki, K. (1991). Antarctic Acanthocephala. In: Wägele, J. W., and Sieg, J. (Eds.), *Synopses of the Antarctic Benthos, Volume 3* (pp. 1-116). Koenigstein, Koeltz Scientific Books.
