# Supplementary material for: Revisiting the genus Bolbosoma Porta, 1908 (Acanthocephala: Polymorphidae): host specificity, phylogeny, and species synonymization
Source: Parasit Vectors. 2025 Sep 24;18:392. doi: 10.1186/s13071-025-07015-3 (PMC12462197; doi:10.1186/s13071-025-07015-3)

Figure S4. Putative secondary structures of the 22 tRNAs identified in the mitochondrial genome of *Bolbosoma vasculosum* (Acanthocephala: Polymorphidae) MZ357087

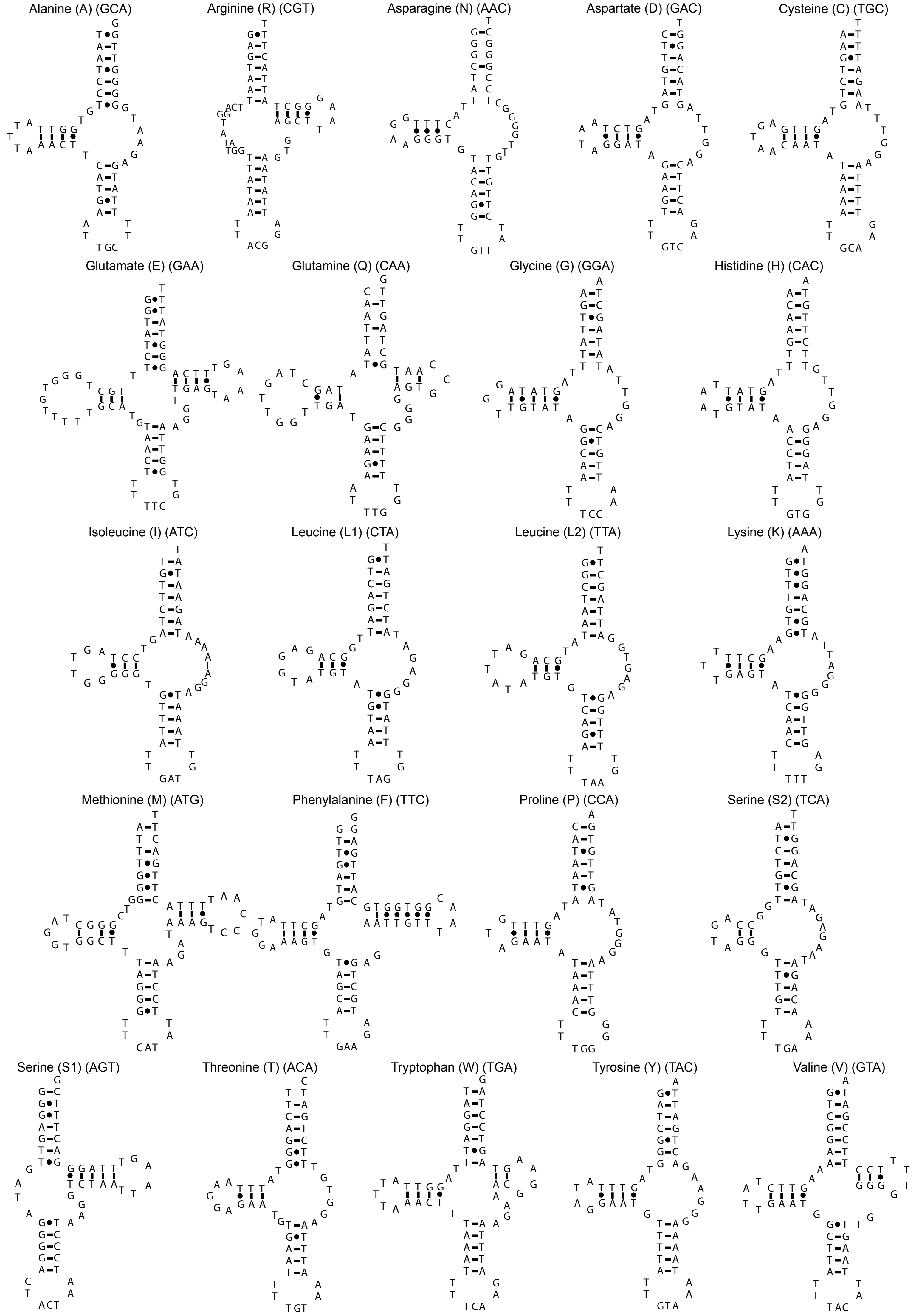

Supplement: Supplementary file 6 — Figure S4. Putative secondary structures of the 22 tRNAs identified in the mitochondrial genome of Bolbosoma vasculosum. [file 13071_2025_7015_MOESM6_ESM.pdf]
