# Supplementary figures and images for: Revisiting the genus Bolbosoma Porta, 1908 (Acanthocephala: Polymorphidae): host specificity, phylogeny, and species synonymization
Source: Parasit Vectors. 2025 Sep 24;18:392. doi: 10.1186/s13071-025-07015-3 (PMC12462197; doi:10.1186/s13071-025-07015-3)

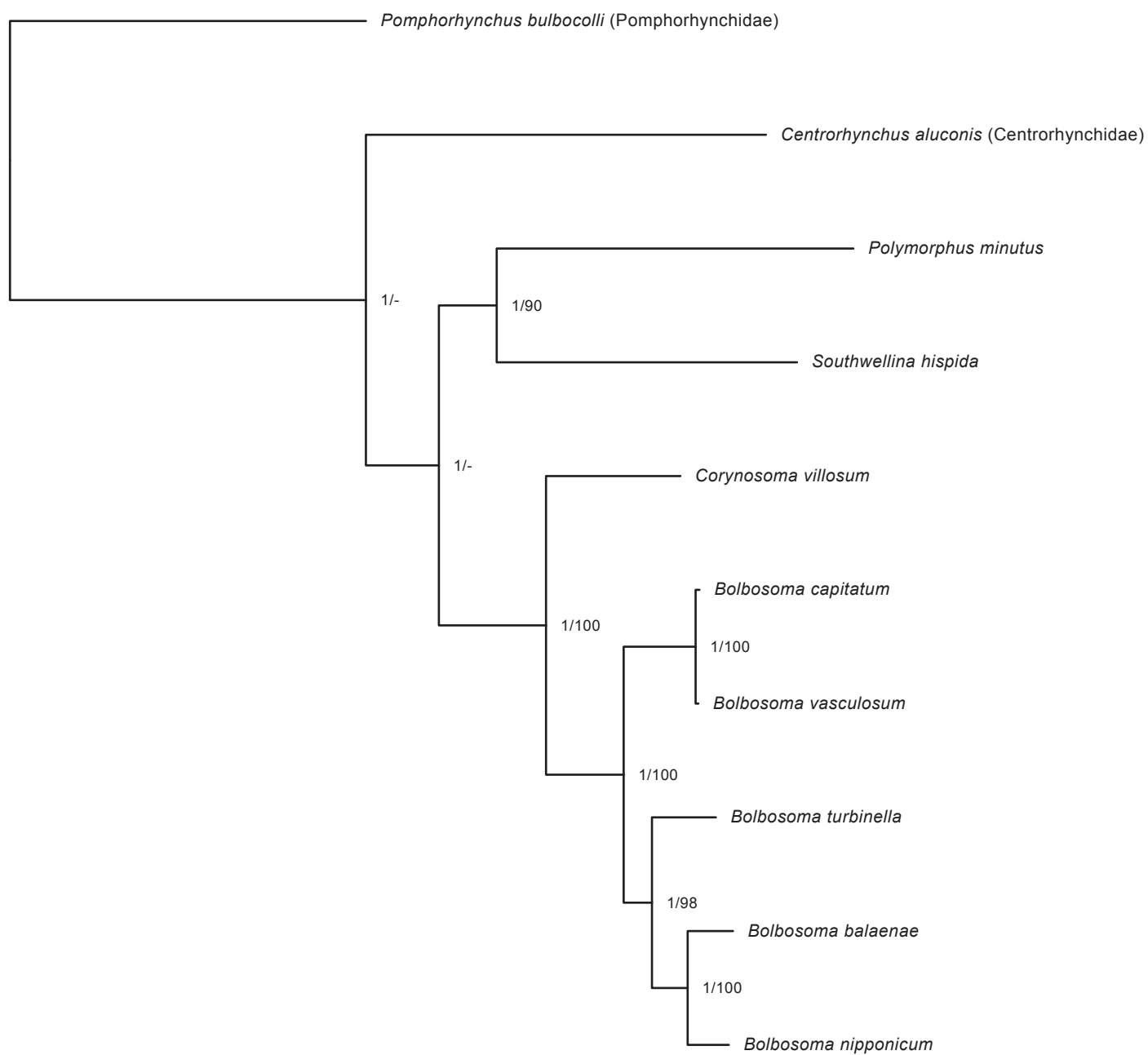

0.07

Supplement: Supplementary file 8 — Figure S5. Phylogenetic relationships of Bolbosoma spp. and related Acanthocephala based on Bayesian and Maximum Likelihood analyses of mitogenome (mixed matrix with protein-coding genes as amino acids) + small subunit ribosomal DNA (ssrDNA) + large subunit ribosomal DNA (lsrDNA) data. Centrorhynchus aluconis (Centrorhynchidae) and Pomphorhynchus bulbocolli (Pomphorhynchidae) were used as outgroups. Posterior probabilities and maximum likelihood bootstrap support values are given for each node. Bootstrap support values < 80% are not shown. Scale bar indicates number of substitutions per site. [file 13071_2025_7015_MOESM8_ESM.pdf]

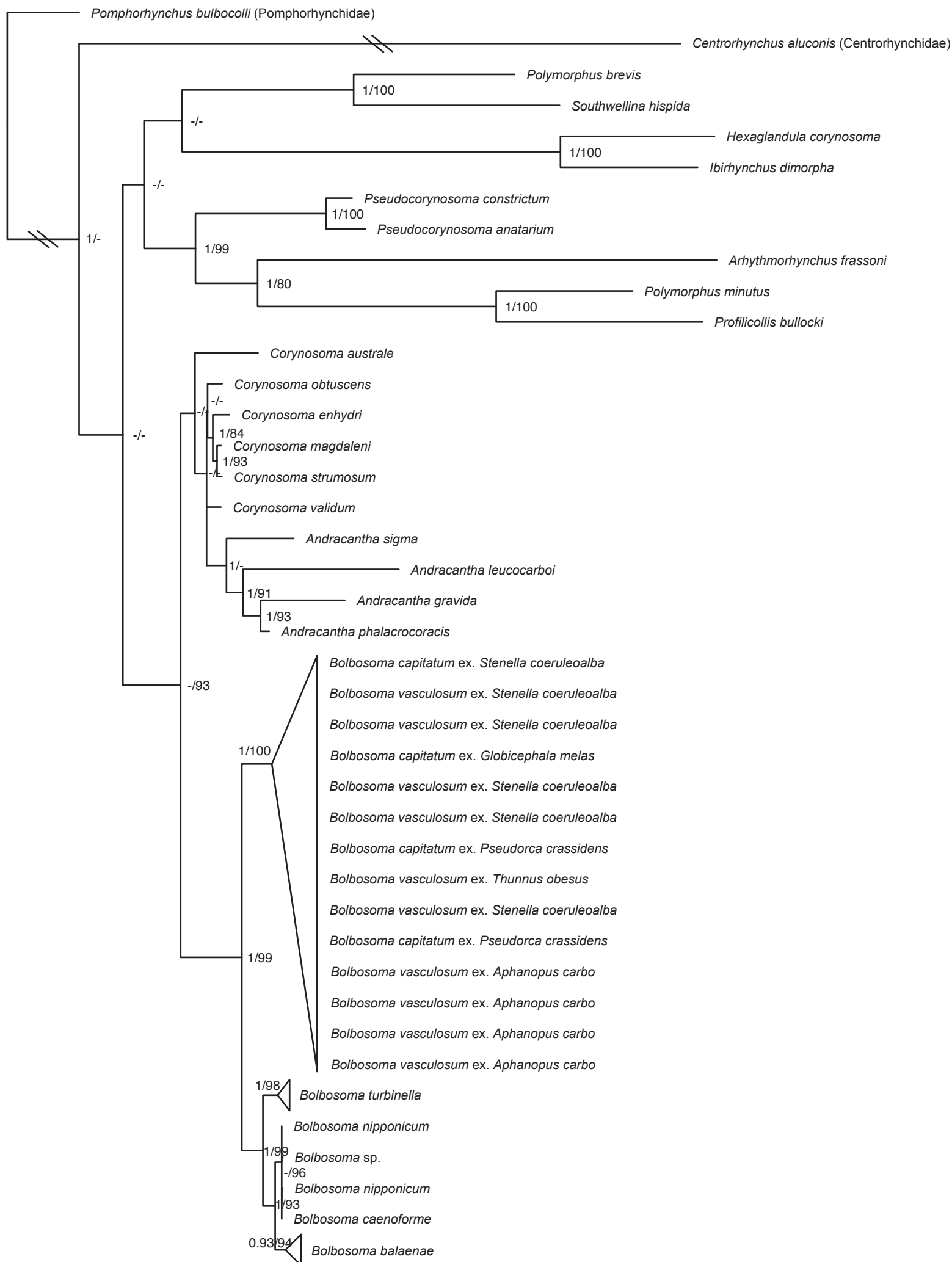

Supplement: Supplementary file 9 — Figure S6. Phylogenetic relationships of Bolbosoma spp. and related Acanthocephala based on Bayesian and Maximum Likelihood analyses of cytochrome c oxidase subunit I (cox1) (as amino acids) + small subunit ribosomal DNA (ssrDNA) + large subunit ribosomal DNA (lsrDNA) data. Centrorhynchus aluconis (Centrorhynchidae) and Pomphorhynchus bulbocolli (Pomphorhynchidae) were used as outgroups. Posterior probabilities and maximum likelihood bootstrap support values are given for each node. Posterior probabilities < 0.9 and bootstrap support values < 80% are not shown. Scale bar indicates number of substitutions per site. [file 13071_2025_7015_MOESM9_ESM.pdf]

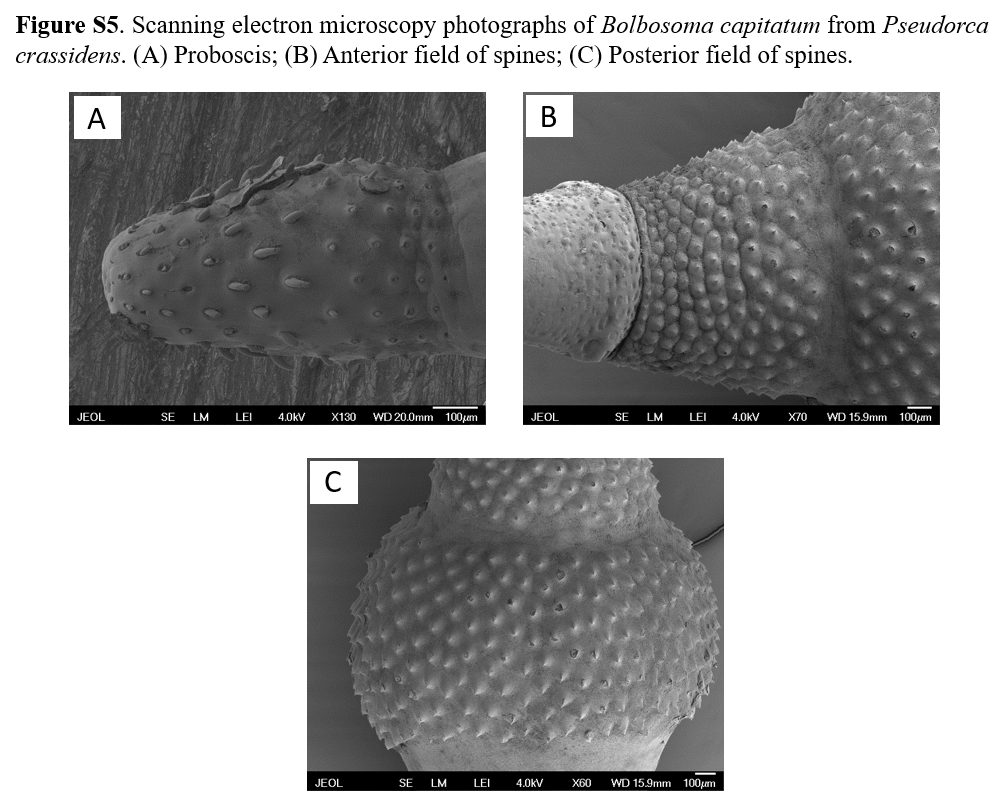

Supplement: Supplementary file 10 — Figure S7. Scanning electron microscopy photographs of Bolbosoma capitatum from Pseudorca crassidens. [file 13071_2025_7015_MOESM10_ESM.png]
